# A cell cycle-dependent BRCA1–UHRF1 cascade regulates DNA double-strand break repair pathway choice

**DOI:** 10.1038/ncomms10201

**Published:** 2016-01-05

**Authors:** Haoxing Zhang, Hailong Liu, Yali Chen, Xu Yang, Panfei Wang, Tongzheng Liu, Min Deng, Bo Qin, Cristina Correia, Seungbaek Lee, Jungjin Kim, Melanie Sparks, Asha A. Nair, Debra L. Evans, Krishna R. Kalari, Pumin Zhang, Liewei Wang, Zhongsheng You, Scott H. Kaufmann, Zhenkun Lou, Huadong Pei

**Affiliations:** 1 School of Life Sciences, Southwest University, Chongqing 400715, China; 2 State Key Laboratory of Proteomics, Beijing Proteome Research Center, Beijing Institute of Radiation Medicine, Beijing 100850, China; 3 Division of Oncology Research, Mayo Clinic, Rochester, MN 55905, USA; 4 Department of Cell Biology and Physiology, Washington University, St Louis, Missouri 63130, USA; 5 BSI-Genetics & Bioinformatics, Mayo Clinic, Rochester, Minnesota 55905, USA; 6 Department of Molecular Physiology and Biophysics, Baylor College of Medicine, Houston, Texas 77030, USA; 7 Molecular Pharmacology and Experimental therapeutics, Mayo Clinic, Rochester, Minnesota 55905, USA

## Abstract

BRCA1 is an important mediator of the DNA damage response, which promotes homologous recombination (HR) and antagonizes 53BP1-dependent non-homologous end joining in S/G2 phase. But how this is achieved remains unclear. Here, we report that the E3 ubiquitin ligase UHRF1 (Ubiquitin-like, with PHD and RING finger domains 1) directly participates in the interplay between BRCA1 and 53BP1. Mechanistically, UHRF1 is recruited to DNA double-strand breaks (DSBs) by BRCA1 in S phase, which requires the BRCT domain of BRCA1 and phosphorylated Ser674 of UHRF1. Subsequently, UHRF1 mediates K63-linked polyubiquitination of RIF1, and results in its dissociation from 53BP1 and DSBs thereby facilitating HR initiation. Thus, UHRF1 is a key regulator of DSB repair choice, which is separate from its role in heterochromatin formation and epigenetic regulator.

## Abstract (web summary)

BRCA1 is a key regulator of DNA double-strand break repair, functioning to promote homologous recombination and repress non-homologous end-joining. Here the authors show that the ubiquitin ligase UHRF1 is recruited to breaks by BRCA1, where it targets RIF1 and thereby facilitates recombination.

## Graphical abstract

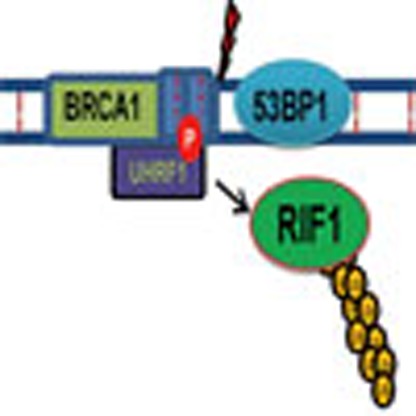

DNA double-strand breaks (DSBs) pose a serious hazard to cell viability and genome stability. Proper repair of chromosomal DSBs is critical for maintaining genome stability and preventing tumorigenesis. In eukaryotic cells, non-homologous end joining (NHEJ) and homologous recombination (HR) are two key pathways that mediate the repair of DSBs^1^,^2^,^3^,^4^. Initiation of these processes is tightly regulated, and aberrant pathway activation results in genomic instability^5^,^6^,^7^,^8^,^9^. NHEJ repairs DSBs by the religation of broken DNA ends. Without the use of a homologous template to guide repair, NHEJ is considered error prone and mutagenic^10^. HR is considered an error-free mechanism for DSB repair that employs homologous sequence in the sister chromatid as a template to prime repair synthesis and restore chromosome integrity^3^.

The choice between HR and NHEJ is tightly regulated during the cell cycle^2^,^3^,^11^,^12^,^13^. NHEJ functions throughout the cell cycle, whereas HR primarily occurs in S and G2 phases of the cell cycle when a sister chromatid is present. In G1 cells, NHEJ is the only choice because the sister chromatid is unavailable and DNA end resection is suppressed. In S-phase cells, DSB end resection leads to initiation of HR. Consequently, repair pathway choice switches from NHEJ to HR. 53BP1 and BRCA1 play antagonizing roles during this process^6^,^14^,^15^,^16^. Recent studies revealed that 53BP1 blocks BRCA1 DSB relocation and promotes NHEJ in G1-phase cells through the recruitment of its downstream effector RIF1 (refs 14, 15, 16, 17, 18, 19, 20, 21, 22, 23).

In G1 phase, RIF1 accumulates at DSB sites by interacting with phosphorylated 53BP1 to prevent 5′ end resection and promote NHEJ. In the absence of RIF1, DSBs are hyperresected and cells show G1-specific hypersensitivity to ionizing radiation^18^,^19^,^20^,^21^,^22^. Conversely, in S-phase cells, RIF1 is removed from DSB sites in a BRCA1-dependent manner, which is critical for the initiation of HR. In this context, BRCA1 facilitates HR by removing RIF1 from DSBs in S phase. However, the underlying mechanism remains unclear, as conflicting results were reported as to the role of CtIP in this process^18^,^19^.

UHRF1 (Ubiquitin-like, with PHD and RING finger domains 1; also called Np95 and ICBP90) has been shown to be an important epigenetic regulator that bridges DNA methylation and chromatin modification^24^,^25^,^26^,^27^,^28^,^29^. Interestingly, several studies have also revealed that disruption of UHRF1 function results in hypersensitivity to DNA damage^30^,^31^,^32^,^33^,^34^,^35^, suggesting a critical role for UHRF1 in the maintenance of genome stability. However, how UHRF1 functions in the DNA repair choice remains unclear.

Here, we report that UHRF1 functions downstream of BRCA1 and is important for BRCA1-mediated removal of RIF1 from DSBs. Following DNA damage, UHRF1 is recruited to the DSBs in S phase via an interaction between the BRCT domain of BRCA1 and phosphorylated UHRF1. UHRF1, in turn, catalyses the K63-linked polyubiquitination of RIF1, decreasing the interaction between RIF1 and 53BP1 and promoting RIF1 dissociation from DSB sites. Consequently, the blockade of BRCA1 foci by RIF1 is released and HR occurs in S phase. These results suggest that UHRF1 is a critical effector of BRCA1 that promotes HR.

## Results

### UHRF1 interacts with BRCA1 and is important for DDR

**Figure 1:**
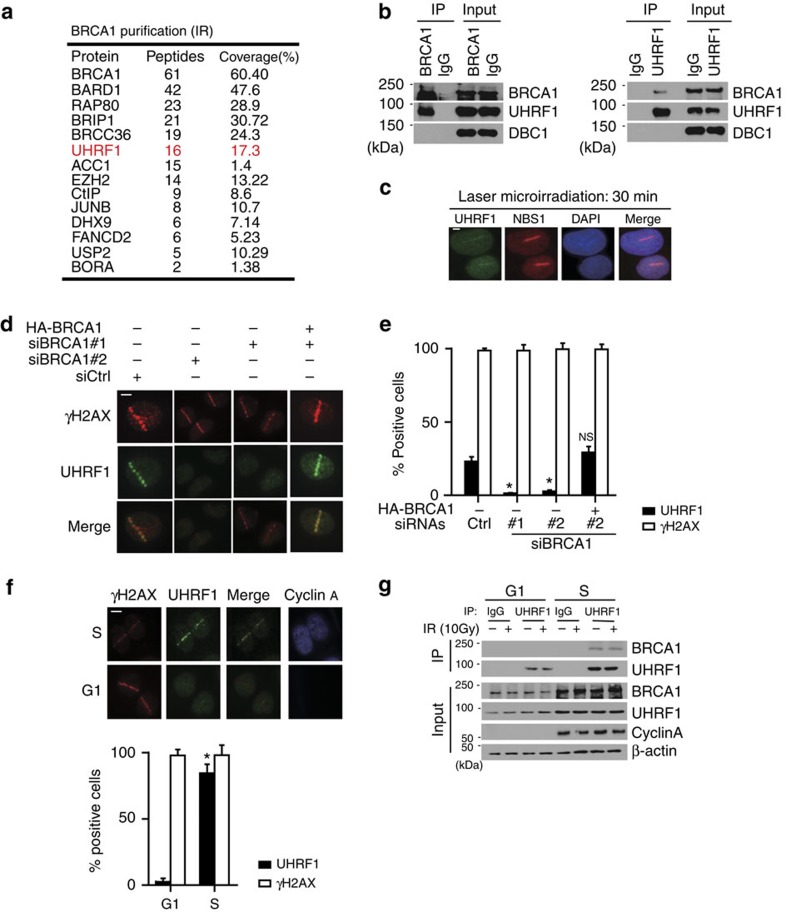
UHRF1 interacts with BRCA1 and is recruited to DNA damage sites by BRCA1 in S phase. (**a**) Tandem affinity purification was performed using 293T cells stably expressing Flag-tagged BRCA1. The major hits from mass spectrometry analysis were shown in the table. (**b**) Reciprocal Co-IP between UHRF1 and BRCA1 in HeLa cells was performed. DBC1 was blotted as a negative control. (**c**) UHRF1 translocate to DSBs following DNA damage. U2OS cells were subjected to laser microirradiation to generate DSBs in a line pattern. Cells were then fixed after 30 min and immunostained with the indicated antibodies. Scale bar, 10 μm. (**d**,**e**) UHRF1 accumulation at DSBs requires BRCA1. U2OS cells were transfected with indicated constructs, then subjected to laser microirradiation to generate DSBs in a line pattern. Cells were then fixed and immunostained with the indicated antibodies (**d**) Representative micrographs (**e**) Quantification for (**d**), for each condition, 200 cells were counted. All the experiments were repeated three times biologically. Error bars represent the mean±s.d. of three biological triplicates. UHRF1 recruitment positive cell percentage compared with control group *P<0.05. NS: no siginficant difference. Scale bar, 10 μm. (**f**) UHRF1 recruitment to DSBs mainly occurs in S phase. U2OS cells were synchronized and subjected to laser microirradiation to generate DSBs in a line pattern. Cells were then fixed and immunostained with the indicated antibodies. Upper: representative micrographs; Lower: quantification. For each condition, 200 cells were counted. *P<0.05. NS: no siginficant difference. Scale bar, 10 μm. (**g**) The UHRF1–BRCA1 interaction is cell cycle regulated. HeLa cells were synchronized at G1 or S phase. Cell lysates were prepared and subjected to immunoprecipitation and immunoblot with the indicated antibodies.

To search for downstream effectors of BRCA1 involved in HR, BRCA1 purification was performed using HEK 293T cells stably expressing SBP-tagged BRCA1. Before or after exposure to ionizing radiation (IR), chromatin-associated BRCA1 complexes were isolated and subjected to mass spectrometry analysis. A number of known BRCA1-associated proteins were co-purified with BRCA1, including BARD1, CtIP, RAP80, ACC1 and BRCC36 (Fig. 1a; Supplementary Fig. 1a). Interestingly, we also identified UHRF1, a well-characterized epigenetic regulator, as a BRCA1-associated protein both before and after DNA damage. To confirm this interaction, we performed reciprocal coimmunoprecipitation (Co-IP) assays with antibody against UHRF1 or BRCA1. As shown in Fig. 1b, endogenous UHRF1 and BRCA1 interact with each other in cells.

Next, we asked whether UHRF1 is recruited to DNA damage sites. UHRF1 was observed at DSBs induced by laser irradiation (Fig. 1c), which appeared at early time points and sustained at DSBs for hours (Supplementary Fig. 1b). To investigate whether BRCA1 is important for UHRF1 recruitment, we knocked down BRCA1 by two different siRNAs, and examined UHRF1 recruitment. As shown in Fig. 1d,e and Supplementary Fig. 1c, UHRF1 recruitment to DSBs could be markedly blocked by BRCA1 depletion. Reconstituting cells with BRCA1 restored UHRF1 recruitment, suggesting the accumulation of UHRF1 to DSBs requires BRCA1. Surprisingly, we noticed that the recruitment of UHRF1 to DSBs did not occur in every cell (Fig. 1d; Supplementary Fig. 1b). Given the recent findings of cell phase-specific BRCA1 recruitment^19^,^20^,^21^, we reasoned that UHRF1 might behave similarly to BRCA1 on DSBs accumulation. Consistent with our hypothesis, in synchronized cell populations, the recruitment of UHRF1 to DSBs was mainly observed in S-phase cells (Fig. 1f). Since UHRF1 focus formation requires BRCA1, we next tested whether the association between BRCA1 and UHRF1 is regulated during cell cycle progression. Indeed, we found that an S-phase-specific interaction between BRCA1 and UHRF1 (Fig. 1g).

### BRCA1 recruits UHRF1 to DSBs through BRCT domain

**Figure 2:**
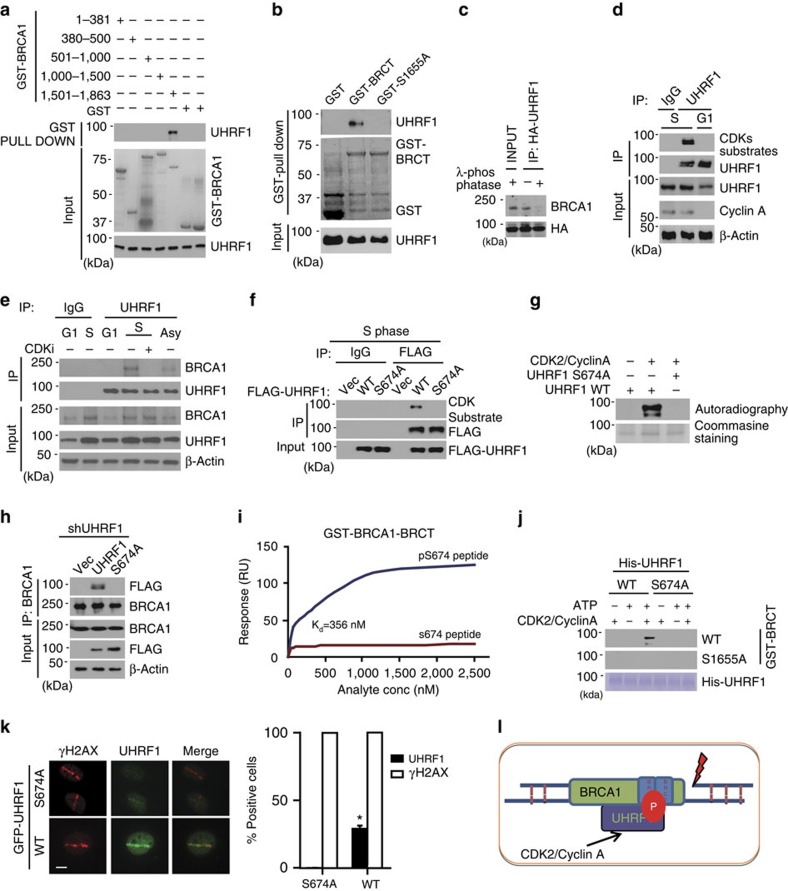
BRCA1 recruits UHRF1 to DSBs through BRCT domain. (**a**) GST-pull-down assay of UHRF1 using the indicated GST fusion proteins. (**b**) GST-pull-down assay of UHRF1 using wild-type BRCA1 BRCT domain or S1655A mutant. (**c**) UHRF1’s association with BRCA1 depends on phosphorylation. HEK 293T cells transfected with HA-UHRF1 were treated as indicated. The UHRF1–BRCA1 interaction was examined. (**d**) UHRF1 was phosphorylated by CDKs in S phase. HeLa cells were synchronized, and UHRF1 phosphorylation was examined with indicated antibodies. (**e**) CDKs inhibitor abolishes the interaction between UHRF1 and BRCA1 in S phase. HeLa cells were synchronized and treated with Roscovitine. UHRF1 phosphorylation was examined as indicated. (**f**) UHRF1 Ser674 was phosphorylated in S phase. HEK 293T cells transfected with the indicated constructs were synchronized at S phase, UHRF1 phosphorylation was examined. vec: empty vector. (**g**) UHRF1 is phosphorylated by CDK2/cyclin A. *In vitro* kinase assay was performed with CDK2/cyclin A using recombinant wild-type UHRF1 or UHRF1-S674A mutant as substrates. (**h**) Ser674 was important for UHRF1–BRCA1 interaction. HEK 293T cells stably expressing UHRF1 shRNA were transfected with indicated constructs. The BRCA1–UHRF1 interaction was examined following synchronization to S phase. (**i**) GST-BRCA1-BRCT fusion protein (17 nM to 2.5 μM) was passed over BIAcore chip (SA chip) surfaces immobilized with control peptide or phosphorylated S674 peptide. Resonance units were measured by BIAcore TP200. (**j**) Phosphorylated UHRF1 directly bind the BRCT domain of BRCA1. His-UHRF1 protein was subjected to CDK2/cyclin A phosphorylation or mock treatments *in vitro*, and resolved by SDS–PAGE. The binding of UHRF1 to the BRCT domain was analysed by Far-western blots. (**k**) Ser674 phosphorylation is important for UHRF1 accumulation at DSBs. U2OS cells were transfected with wild-type GFP-UHRF1 or S674A mutant. DSBs accumulation was monitored following laser microirradiation. For each sample, 200 cells were counted. Error bars represent the mean±s.d. of biological triplicates. Left: representative images; Right: quantification of positive signal. Error bars represent the mean±s.d. of biological triplicates. UHRF1 recruitment positive cell percentage compared with control group: *P<0.05. Scale bar, 10 μm. (**l**) Schematic model illustrating Figs 1–2.

To determine how BRCA1 recruits UHRF1 to DSBs, we mapped the domains of BRCA1 that interact with UHRF1. UHRF1 could only be pulled down by the C-terminal region (GST-BR4: AA1501-1863) of BRCA1 containing the BRCT domain (Fig. 2a). Further, we observed that UHRF1 could be pull-down by the purified GST-BRCT (wild type) domain of BRCA1, but not by the BRCT (S1655A) mutant that is defective for binding to phospho-Ser/Thr motifs (Fig. 2b). This indicates that the BRCT activity of BRCA1 is required for the association with UHRF1.

Since the BRCT domain is well known for binding the phosphorylated partner to facilitate DNA damage response signalling^36^,^37^. We hypothesized that BRCA1 interacts with phosphorylated UHRF1 through BRCT domain. To test this, we performed Co-IP assay with or without lambda phosphatase (λ PPase) treatment. As shown in Fig. 2c, λ PPase treatment abolished the interaction between UHRF1 and BRCA1, suggesting that phosphorylation is critical for the UHRF1–BRCA1 interaction. Considering that the interaction between BRCA1 and UHRF1 mainly occurred in S phase (Fig. 1g), it is possible that the S-phase-specific phosphorylation of UHRF1 is important for binding BRCA1. Consistently, we detected that UHRF1 could be recognized by specific antibody against phosphorylated CDK substrates in S phase (Fig. 2d). Moreover, the interaction between UHRF1 and BRCA1 in S phase was disrupted in cells treated with a CDK inhibitor (Roscovitine), even though BRCA1 and UHRF1 levels did not decrease by Roscovitine treatment (Fig. 2e). These results suggest the UHRF1 phosphorylation by CDKs is prerequisite for its interaction with BRCA1.

Sequence analysis showed that there are two residues, serine 652 and serine 674 (UHRF1 has two isoforms, in this study, we used the UHRF1 isform 2: NP_037414.3), that resemble the consensus motif (K/R)(S*)PX(K/R) recognized by CDKs. A previous study showed that S661 of UHRF1 of isoform 1 (corresponding to S674 in isoform 2) is phosphorylated by CDK2/cyclin A and the corresponding serine in the zebrafish homologue (Serine 648) was also phosphorylated^38^. Serine 639 in UHRF1 isoform 1 (corresponding to Serine 652 of isoform 2) was also reported to be phosphorylated by CDK1/cyclin B in M phase^39^. We mutated these two sites separately and observed that mutation of S674A, but not S652A, abolished the recognition by the CDKs substrate antibody in S phase (Fig. 2f). Further, through *in vitro* kinase assay, we confirmed that CDK2/cyclin A, which is well known active CDK complex functions specifically in S phase, could directly phosphorylate UHRF1 on serine 674 (Fig. 2g), suggesting that S674 is phosphorylated by CDK2. Importantly, UHRF1-S674A mutant disrupted the association between UHRF1 and BRCA1 (Fig. 2h).

To confirm that the BRCA1 BRCT domain binds phosphorylated S674, we synthesized peptides corresponding to residues 667 to 681 of UHRF1, which contained either phosphorylated or unphosphorylated S674. Using Surface Plasmon Resonance (SPR) assay, we confirmed the direct interaction between the pS674 peptide and the BRCA1 BRCT domain. The BRCA1 BRCT bound tightly to the phosphorylated peptide with little binding to unphosphorylated peptide (Fig. 2i; Supplementary Fig. 1d). The dissociation constant (*K*d) was calculated as 356 nM. Further, to demonstrate the direct interaction between BRCA1 and CDK2-phosphorylated UHRF1, we performed Far-Western assay. As shown in Fig. 2j, only *in vitro* phosphorylated wild-type (WT) UHRF1 could interact with the WT BRCT domain of BRCA1, while unphosphorylated WT UHRF1 or the S674A mutant could not. The BRCT S1655A mutant also could not interact with phosphorylated UHRF1. Moreover, the S674A mutant showed defective recruitment to DSBs following laser microirradiation (Fig. 2k). These results suggest that UHRF1 Serine 674 phosphorylation by CDK2/cyclin A is required for its binding to BRCA1 and subsequent accumulation at DSB sites (Fig. 2l). It is interesting to note that, compared with Abraxas, CtIP or BACH1, the BRCT binding site (Serine 674) on UHRF1 does not fit with the consensus binding motif of the BRCA1 BRCT domain^36^,^37^. Therefore, S674 of UHRF1 is a noncanonical binding site of the BRCT domain of BRCA1, suggesting that other BRCA1 BRCT ligands might exist.

### UHRF1 function in DNA repair require its E3 ligase activity

**Figure 3:**
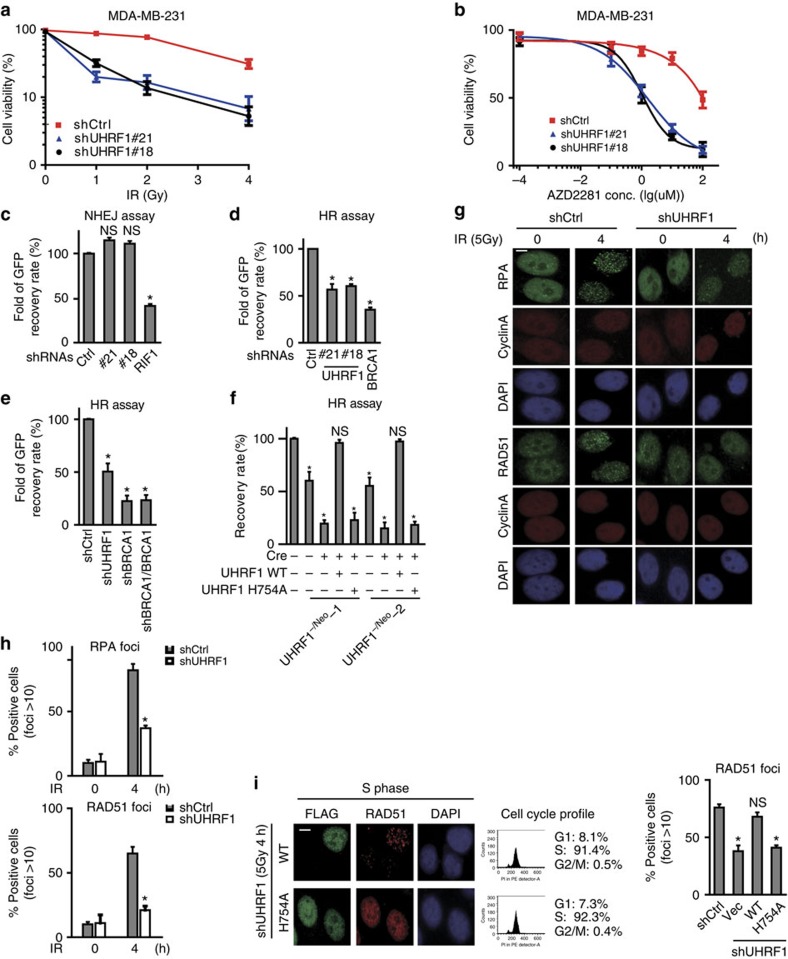
UHRF1 is involved in DNA repair through its E3 ligase activity. (**a**) MDA-MB-231 cells stably expressing the indicated shRNAs were irradiated. Radiation sensitivity of cells was determined by colony formation assays. Data were presented as mean±s.d. of three biological triplicates. (**b**) MDA-MB-231 cells stably expressing indicated shRNAs were treated with increasing doses of PARP inhibitor (AZD2281). MTS assay was performed to determine the surviving fraction. Data presented as mean±s.d. of three biological triplicates. (**c**–**e**). HEK293 cells integrated with NHEJ or HR reporter were transfected with the indicated shRNAs and subjected to the NHEJ assay (**c**) or HR assay (**d**,**e**) as described in Methods. Data presented as mean±s.d. of three biological triplicates.. Positive cell percentage compared with control group. *P<0.05. NS: no siginficant difference. (**f**). UHRF1^flox/hypo^ cells transfected with HR reporter were treated as indicated. HR assay were performed as described in Methods. Data presented as mean±s.d. of three biological triplicates. Positive cell percentage compared with control group. *P<0.05. NS: no siginficant difference. (**g**,**h**). UHRF1 regulates RPA and RAD51 IRIF. RPA and Rad51 foci formation were examined in synchronized HeLa cells stably expressing control or UHRF1 shRNA following irradiation (5 Gy). Cyclin A was stained as a cell cycle marker. (**g**) Representative micrographs. (**h**) Quantification data (foci>10 per cell). For each sample, randomly selected S phase cells (*n*=400) were counted. Data presented as mean±s.d. of three biological triplicates. Positive cell percentage compared with control group. *P<0.05. Scale bar, 10 μm. (**i**). HeLa cells stably expressing UHRF1 shRNA (targeting 3′-UTR) were reconstituted with the indicated constructs and synchronized in S phase. RAD51 foci were examined following irradiation (5 Gy 4 h). left: Representative micrographs. Right: Quantification of the positive cells (foci>10 per cell). For each sample, 400 randomly selected cells were counted. Data presented as mean±s.d. of three biological triplicates. Positive cell percentage compared with control group. *P<0.05. NS: no siginficant difference, Scale bar, 10 μm.

UHRF1 was shown to be important for the DNA damage response, although the underlying mechanism remains unclear^30^,^33^,^40^. Consistent with these findings, we found that depletion of UHRF1 sensitized cells to IR in cancer cells (Fig. 3a; Supplementary Fig. 1e). In addition, similar to BRCA1 depletion, UHRF1 depletion rendered cells hypersensitive to PARP inhibitor (AZD2281) (Fig. 3b; Supplementary Fig. 1e), implying UHRF1’s important role in the HR pathway.

Next, we examined how UHRF1 promotes DNA repair using integrated reporter assays for HR and NHEJ^41^,^42^,^43^. We observed a moderate elevation in NHEJ efficiency (Fig. 3c; Supplementary Fig. 1f). Importantly, UHRF1 depletion led to significantly compromised HR (Fig. 3d; Supplementary Fig. 1g). BRCA1 knockdown showed much more significant HR deficiency. However, we did not observe significant further defect in HR when we co-depleted BRCA1 and UHRF1 (Fig. 3e; Supplementary Fig. 1h), suggesting that UHRF1 and BRCA1 are epistatic and UHRF1 is an important downstream factor in BRCA1-mediated HR regulation. Further, using hypomorphic mutants (UHRF1^−/Neo^-1 and UHRF1^−/Neo^-2) in which one UHRF1 allele was inactivated and the other was rendered hypomorphic^34^, we also detected drastically HR repair defect result from UHRF1 deficiency, strongly suggesting that UHRF1 is important for proper HR repair (Fig. 3f; Supplementary Fig. 1i,j). Taken together, UHRF1 plays a role in regulating DSB repair choice, in which HR is the major targeted pathway.

In agreement with this, we observed that UHRF1 depletion resulted in sharply decreased RPA recruitment and Rad51 loading to DSBs (Fig. 3g,h; Supplementary Fig. 2c), without affecting the upstream DNA repair factors (MDC1, RNF8 and RNF168) and ubiquitination signals (FK2); (Supplementary Fig. 2d–f). In addition, we tested whether UHRF1 deficiency affects the interaction between BRCA1 and other BRCA1 binding partners. FANCJ, CtIP and Abraxas, all of which have been reported to bind the BRCA1 BRCT domain, showed intact interaction with either BRCA1 or the BRCA1 BRCT domain following UHRF1 depletion (Supplementary Fig. 2a,b). These results support the direct and important role of UHRF1 downstream of BRCA1 regulating DNA repair pathway choice, particularly in HR regulation.

Since UHRF1 is an E3 ubiquitin ligase^44^, we asked whether its ubiquitin ligase activity is required to regulate HR. We stably knocked down UHRF1 in cells using shRNA targeting the 3′-UTR region of UHRF1, and reconstituted cells with WT UHRF1 or UHRF1-H754A mutant (disabled UHRF1 E3 ubiquitin ligase activity)^44^,^45^. As shown in Fig. 3i, WT UHRF1, but not the H754A mutant, restored Rad51 foci in UHRF1-depleted cells after IR. These results indicate that the E3 ligase activity of UHRF1 is required for its function in HR regulation.

In our study, we did not detect a remarkable cell cycle alteration caused by stably depleting UHRF1 (Supplementary Figs 2g and 4a,b). This is consistent with one previous report^46^ but differs from some previous studies^32^,^40^. A recent study^34^ also showed that while knockout of UHRF1 results in severe proliferation defect, cells with hypomorphic mutants that significantly reduced UHRF1 levels maintain normal growth. Therefore, in our experimental setting, the defect on DNA repair by UHRF1 depletion did not result from an indirect effect of dysregulated cell cycle progression. It is also noteworthy that UHRF1 was shown to regulate BRCA1 expression^47^, but we failed to observe a significant change in BRCA1 levels when we manipulated UHRF1 levels (Supplementary Fig. 2h,i).

### UHRF1 ubiquitinates RIF1

**Figure 4:**
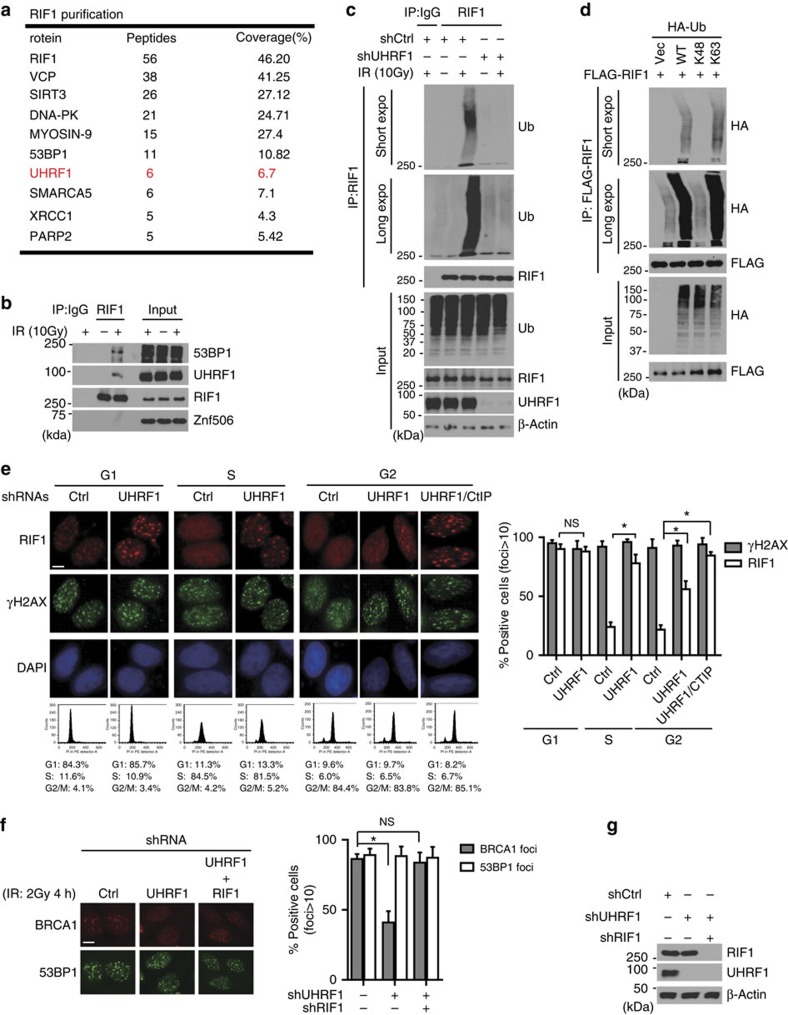
UHRF1 ubiquitinates RIF1. (**a**) Tandem affinity purification of RIF1 was performed using antibody against endogenous RIF1. The major hits from mass spectrometry analysis were shown in the table. (**b**) Endogenous Co-IP between UHRF1 and RIF1 in HeLa cells was performed. 53BP1 and Znf 506 were blotted as positive and negative control respectively. (**c**) UHRF1 ubiquitinates RIF1 *in vivo*. HeLa cells stably expressing control or UHRF1 shRNA were irradiated (10 Gy), and RIF1 ubiquitination was then examined under denaturing conditions 1 h after IR (see Methods). (**d**) UHRF1-mediated K63-linked polyubiquitination of RIF1 *in vivo*. HEK 293T cells were transfected with the indicated constructs. RIF1 ubiquitination was examined as in **c**. (**e**) UHRF1 promotes removal of RIF1 from DSB sites in S phase. HeLa cells stably expressing the indicated shRNAs were synchronized and irradiated. RIF1 and H2AX foci formation were then examined. Left: Representative micrographs. Lower panels: representative cell cycle profiles of synchronized cells. Right: Quantitation of the positive cells (foci>10 per cell) as indicated. For each sample, 600 randomly selected cells were counted. Data presented as mean±s.d. of three biological triplicates. RIF1 foci Positive cell percentage compared with control group. *P<0.05. NS: no siginficant difference, Scale bar, 10 μm. (**f**) HeLa cells expressing the indicated shRNAs were irradiated and immunostained with antibodies as indicated. Left: representative micrographs. Right: quantification of BRCA1 and 53BP1 foci positive cells (foci>10 per cell). For each sample, randomly selected 500 cells were counted. Data presented as mean±s.d. of three biological triplicates. Positive cell percentage compared with control group. *P<0.05. NS: no siginficant difference, Scale bar, 10 μm. (**g**) Immunoblot with indicated antibodies for the cell lysates in **f**.

Our results revealed that BRCA1 interacts with UHRF1 directly and UHRF1 is important for HR. In addition, the ubiquitin ligase activity of UHRF1 is important for DNA repair pathway choice. However, the target(s) of UHRF1 in HR regulation remain unclear. Interestingly, our previous mass spectrometry analysis of RIF1-interacting proteins identified UHRF1 as one of the prominent hits (Fig. 4a). The association of UHRF1 and RIF1, which is induced by DNA damage, was validated by endogenous Co-IP assay (Fig. 4b). Moreover, we observed that the interaction between UHRF1 and RIF1 specifically occurred in S phase (Supplementary Fig. 3a). RIF1 was recently reported to regulate DNA damage repair choice downstream of 53BP1 by antagonizing BRCA1 function^18^,^19^,^20^,^21^,^22^. Since UHRF1 and RIF1 have opposite functions in DNA repair choice regulation, we reasoned that UHRF1 may antagonize RIF1 function through its E3 ligase activity.

To test this hypothesis, we first examined RIF1 ubiquitination following DNA damage. As shown in Fig. 4c, RIF1 was ubiquitinated *in vivo* following DNA damage, which was abrogated in UHRF1-depleted cells, indicating that UHRF1 mediates DNA damage-induced polyubiquitination of RIF1. Interestingly, RIF1 levels were not affected by UHRF1 depletion. In contrast to Lys-48 (K48)-linked polyubiquitin chain, Lys 63 (K63)-linked ubiquitin chains have been implicated in the DNA damage response for signalling transduction rather than proteasome-directed protein degradation. Indeed, we found it was K63 linked, but not K48 linked, polyubiquitin chains were conjugated to RIF1 following DNA damage (Fig. 4d). In agreement with this, DNA damage-induced RIF1 ubiquitination also depended on the presence of UBC13, an E2 Ubiquitin conjugating enzyme catalyzing K63-specific protein ubiquitination *in vivo* (Supplementary Fig. 3b).

Previous studies suggest that RIF1 forms IR-induced foci predominantly in G1 phase and RIF1 foci formation is blocked in S phase by BRCA1. This regulation, which depends on the BRCT domain but not the E3 ligase activity of BRCA1, facilitates the switch of DSB repair mode from NHEJ to HR^18^,^19^,^20^,^21^,^22^. As UHRF1 interacts with BRCA1 in S phase and induces RIF1 ubiquitination after IR, we next asked whether UHRF1-mediated RIF1 ubiquitination was important for the regulation of RIF1 accumulation at DSBs. We monitored the effect of UHRF1 depletion on IR-induced RIF1 focus formation in synchronized cells. Consistent with previous studies, RIF1 foci were blocked in S-phase cells following IR in control cells (Fig. 4e). Importantly, UHRF1 depletion led to formation of RIF1 foci in S phase (Fig. 4e). It has been suggested that CtIP could suppress RIF1 foci formation in S/G2 phase^19^. However, another study showed that CtIP does not affect RIF1 foci in S phase but partially affects RIF1 foci in G2 phase^18^. We found that depletion of UHRF1 significantly restored RIF1 foci in S phase (Fig. 4e). Depletion of either UHRF1 or CtIP partially restored RIF1 foci in G2 phase (Fig. 4e; Supplementary Fig. 3c). Co-depletion of UHRF1 and CtIP almost fully restored RIF1 foci (Fig. 4e). Collectively, these results suggest that UHRF1 is a major factor in suppressing RIF1 foci in S phase and UHRF1 functions together with CtIP in G2 phase to suppress RIF1 accumulation at DSBs. In addition, to further exclude the possible influence of cell cycle profile changes caused by UHRF1 depletion, conditional knockdown of UHRF1 in G1 or S phase cells was conducted. A similar defective S-phase-specific RIF1 foci removal was detected in UHRF1-depleted cells (Supplementary Fig. 3d). These results demonstrate that UHRF1 is required for RIF1 foci resolution in S phase. These results also suggest that UHRF1 and CtIP likely function independently.

We also examined 53BP1 and BRCA1 focus formation in cells depleted of UHRF1. We found that 53BP1 foci formation was not affected by UHRF1 depletion (Fig. 4f). Interestingly, we noticed that BRCA1 foci significantly decreased in UHRF1-depleted cells (Fig. 4f). The defect in BRCA1 foci formation in UHRF1 knockdown cells was rescued by RIF1 codepletion (Fig. 4f,g). These results indicated that the decrease in BRCA1 foci in UHRF1-depleted cells was an indirect effect caused by increased RIF1 accumulation. In addition, the recruitment of upstream factors such as MDC1 and RNF8 was not affected by UHRF1 depletion (Supplementary Fig. 2d–f). We conclude that the removal of RIF1 from DSBs is dependent on UHRF1 in S phase.

### RIF1 ubiquitination require UHRF1 phosphorylation in S phase

**Figure 5:**
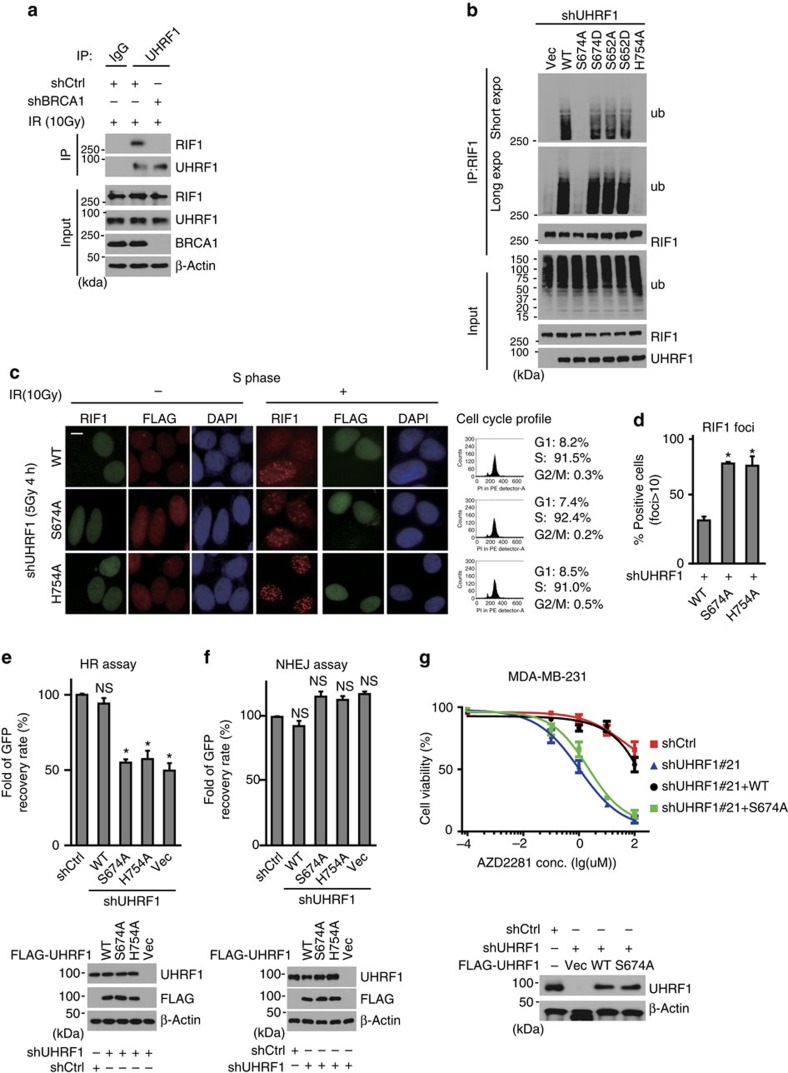
UHRF1 Ser674 phosphorylation is important for RIF1 ubiquitination. (**a**) BRCA1 is required for the UHRF1–RIF1 interaction. HeLa cells expressing the indicated shRNAs were irradiated (10 Gy). The UHRF1–RIF1 interaction was examined 1 h following IR. (**b**) The S674A mutation abolishes UHRF1-mediated RIF1 ubiquitination. HEK 293T cells stably expressing UHRF1 shRNA were reconstituted with the indicated constructs. RIF1 ubiquitination was then examined *in vivo*. (**c**,**d**) Both UHRF1 S674 phosphorylation and UHRF1 E3 ligase activity regulate RIF1 accumulation at DSBs. HeLa cells stably expressing UHRF1 shRNA were reconstituted with the indicated constructs. RIF1 foci formation was then examined. (**c**) Representative micrographs. (**d**) Quantification of the positive cells. For each condition, 500 randomly selected cells were counted. Data presented as mean±s.d. of three biological triplicates. Positive cell percentage compared with WT group. *P<0.05. (**e**,**f**) UHRF1 depleted in HEK293 cells were reconstituted with the indicated constructs were subjected to HR assay (**e**) and NHEJ assay (**f**) as described in Methods. Data presented as mean±s.d. of three biological triplicates. Positive cell percentage compared with control group. *P<0.05. NS: no siginficant difference. (**g**) MDA-MB-231 cells stably expressing UHRF1 shRNA were reconstituted with the indicated constructs. Cell sensitivity to AZD2281 was examined by the MTS assay as in Fig. 3b. Data presented as the mean±s.d. of three biological triplicates.

Since UHRF1 recruitment to DSBs requires BRCA1, we next tested whether the UHRF1–BRCA1 interaction is important for RIF1 regulation. We found that BRCA1 depletion disrupted the association of UHRF1 with RIF1 (Fig. 5a), suggesting that RIF1 was regulated by UHRF1 in a BRCA1-dependent manner. As the S-phase phosphorylation of UHRF1 is required for its BRCA1 interaction, we next explored the functional significance of this S-phase phosphorylation of UHRF1 in RIF1 focus formation. We depleted endogenous UHRF1 and reconstituted cells with WT, UHRF1-S674A or UHRF1 S647D (phosphomimetic mutant). As shown in Fig. 5b, reintroducing WT or the S674D mutant of UHRF1 restored RIF1 ubiquitination induced by DNA damage. Ser 652 is another putative CDKs phosphorylation site, which was previously reported^39^, but we found that it is not required for the association between UHRF1 and BRCA1 (Supplementary Fig. 3e). Reintroducing the S652 mutants (SA or SD) also restored RIF1 ubiquitination. By contrast, reintroducing UHRF1-S674A or the catalytically inactive mutant UHRF1-H754A was unable to restore RIF1 polyubiquitination (Fig. 5b, Supplementary Fig. 3f). Moreover, unlike WT UHRF1, both UHRF1-S674A and UHRF1-H754A were defective in blocking RIF1 accumulation at DSB sites (Fig. 5c,d; Supplementary Fig. 3f). Furthermore, reconstituting UHRF1-deficient cells with WT UHRF1, but not UHRF1-S674A and UHRF1-H754A, restored HR (Fig. 5e,f) and reversed hypersensitivity to PARP inhibitor conferred by UHRF1 depletion (Fig. 5g). Again, we did not detect a remarkable cell cycle alteration in cells expressing WT UHRF1 or S674A mutant (Supplementary Fig. 4a,b). Therefore, the repair defects in cells expressing UHRF1-S674A is not an indirect effect of changes of cell cycle progression.

UHRF1 forms a complex with DNMT1 and regulates epigenetic inheritance during DNA replication^24^,^25^,^28^,^48^. We tested whether DNMT1 gets involved in UHRF1’s function in DNA repair. Notably, DNMT1 did not affect the UHRF1–RIF1 interaction, IR-induced RIF1 ubiquitination, RIF1 foci and HR (Supplementary Fig. 4d,e,g,h). In addition, depletion of both UHRF1 and DNMT1 showed no significant difference in RIF1 focus formation and HR repair efficiency compared with UHRF1 depletion alone (Supplementary Fig. 4g,h). Consistent with established role of UHRF1 in DNA methylation, knockdown of UHRF1 decreased 5mC staining in cells (Supplementary Fig. 4f). Interestingly, reintroducing WT or the S674A mutant restored 5mC staining in UHRF1 knockdown cells (Supplementary Fig. 4f). Therefore, DNA methylation change could not account for HR defect in cells expressing S674A. We further carried out microarray analysis using WT cells, UHRF1 knockdown cells and UHRF1 knockdown cells reconstituted with shRNA-resistant UHRF1-WT or the UHRF1-S674A mutant. We found that knockdown of UHRF1 significantly affected gene expression profiling (Supplementary Fig. 6a,b). However, there was no apparent expression changes in genes involved in DNA repair (Supplementary Data 1; Supplementary Fig. 6a,b). Importantly, cells expressing UHRF1-S674A showed almost identical gene expression profile as WT cells. We further examined histone H3 monoubiquitination mediated by UHRF1, which has been shown to have an important role in coupling DNA methylation and replication^28^. Although UHRF1 knockdown decreased H3 monoubiquitination, WT or the UHRF1-S674A mutant was able to restore histone H3 monoubiquitination (Supplementary Fig. 4c). Therefore, UHRF1-S674A serves as a separation of function mutant that only affect DNA repair, but not several epigenetic functions mediated by UHRF1. All of the above results indicated that UHRF1 is directly involved in HR, and its function in HR is separated from its function in DNA methylation and/or transcription.

### RIF1 ubiquitination is important for its DSBs accumulation

**Figure 6:**
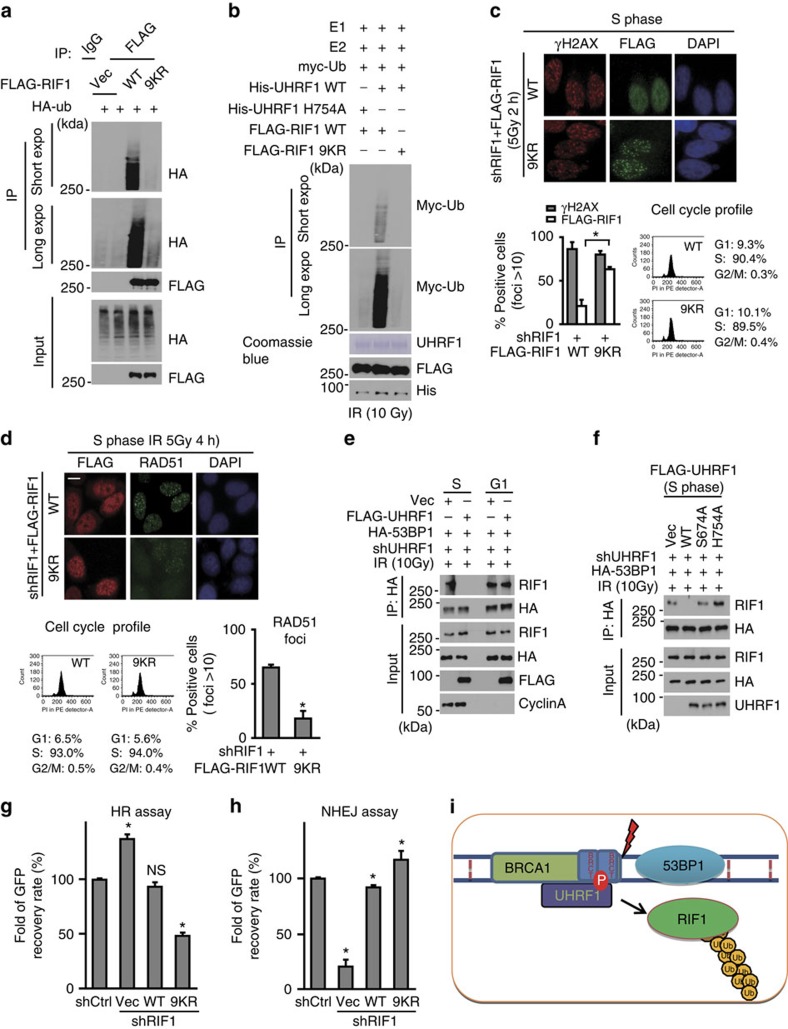
RIF1 ubiquitination by UHRF1 is important for its accumulation at DSB sites. (**a**). Abrogation of RIF1 ubiquitination. HEK 293T cells stably expressing RIF1 shRNA were reconstituted with the indicated constructs. *In vivo* ubiquitination assay was performed as described in Methods. (**b**) UHRF1 ubiquitinates RIF1 *in vitro*. An *in vitro* ubiquitination assay was performed using recombinant UHRF1, ubiquitin, UBC13/Mms2 and UBE1 (See Methods). (**c**) Ubiquitination of RIF1 is required for its recruitment to the DSB sites. HeLa cells stably expressing RIF1 shRNA were reconstituted with the indicated constructs and synchronized. Flag-RIF1 focus formation was then examined following irradiation (5 Gy) as indicated. Upper: Representative micrographs. Lower: quantification of the positive cells (foci>10 per cell) and cell cycle profile. For each sample, 600 randomly selected cells were counted. Data presented as mean±s.d. of three biological triplicates. RIF1 foci Positive cell percentage compared between WT and 9KR group. *P<0.05. (**d**) Ubiquitination of RIF1 is required for RAD51 accumulation at DSBs. HeLa cells stably expressing RIF1 shRNA were reconstituted with the indicated constructs. Rad51 foci formation was examined 4 h following irradiation (5 Gy). Upper: Representative micrographs; Lower: Quantification of the positive cells (foci>10 per cell). For each sample, 600 randomly selected cells were counted. Data presented as mean±s.d. of three biological triplicates. *P<0.05. (**e**) HEK 293T cells stably expressing UHRF1 shRNA were transfected with the indicated constructs and synchronized at G1 or S phase, and 53BP1 and RIF1 interaction was examined. (**f**) HEK 293T cells stably expressing UHRF1 shRNA were reconstituted with the indicated constructs. 53BP1 and RIF1 interaction was examined following synchronization at S phase. (**g**,**h**) Ubiquitination of RIF1 is required for its function in DSB repair. RIF1 was depleted in HEK 293 cells integrated with HR or NHEJ reporter. Cells were reconstituted with the indicated constructs and subjected to HR assay (**g**) and NHEJ assay (**h**) as described in the Methods. Data presented as mean±s.d. of three biological triplicates. Positive cell percentage compared with control group. *P<0.05. NS: no siginficant difference. (**i**) Schematic model illustrating Figs 4.

To explore the importance of RIF1 ubiquitination for its accumulation at DSBs, we mapped the ubiquitination sites in RIF1. Through mass spectrometry analysis and public available database search, we identified nine lysine residues as the potential ubiquitination sites of RIF1. Using a site-specific mutagenesis strategy, we generated RIF1 mutants with all of the nine lysine residues mutated to arginine (Supplementary Fig. 5a). WT or mutant RIF1 was reconstituted in RIF1-depleted cells and RIF1 ubiquitination was examined. As shown in Fig. 6a, mutation of all nine lysine residues (9KR) decreased most RIF1 ubiquitination following IR, suggesting these nine residues are major RIF1 ubiquitination sites following IR. This result, together with the results showing UHRF1 depletion abolished RIF1 ubiquitination, suggests that these nine residues are major UHRF1 ubiquitination sites. Through *in vitro* ubiquitination assay, we confirmed that WT RIF1, but not the 9KR mutant, could be directly ubiquitinated by UHRF1 *in vitro* (Fig. 6b).

Given that UHRF1 regulates RIF1 ubiquitination and RIF1 disassociation from DSBs in S phase, we predicted that the 9KR mutant would be retained at DSBs in S phase. In agreement with our prediction, the 9KR mutant formed damage-induced foci in S phase, in sharp contrast to WT RIF1 (Fig. 6c). Additionally, Rad51 focus formation was defective in cells expressing RIF1 9KR (Fig. 6d), which phenocopied UHRF1 downregulation (Fig. 3g).

We also observed that RIF1 ubiquitination occurred in S phase and was abolished in 53BP1-depleted cells (Supplementary Fig. 5b,c). In addition, we found that RIF1 dissociated from 53BP1 in S phase (Fig. 6e). Further, reconstituting cells with either S674A or H754A abrogated the dissociation of RIF1 from 53BP1 in S phase (Fig. 6f). Thus, one possible mechanism by which UHRF1 ubiquitinates RIF1 and regulates its DSBs accumulation is through affecting the RIF1–53BP1 interaction. 53BP1 is essential to recruit RIF1 to DSBs^18^,^19^,^20^. Our results showed that disrupting either UHRF1 or RIF1 recruitment to DSBs abolished RIF1 ubiquitination (Fig. 5b; Supplementary Fig. 5b,c), suggesting that both UHRF1 and RIF1 are needed to localize at DSBs for RIF1 ubiquitination and that RIF1 is ubiquitinated by UHRF1 at DSBs. Finally, we found that the DNA repair defect in HR caused by RIF1 deletion could be rescued by wild-type RIF1 rather than the 9KR mutant (Fig. 6g,h; Supplementary Fig. 5d–f), supporting the important role of RIF1 ubiquitination in DNA repair choice (Fig. 6i).

**Figure 7:**
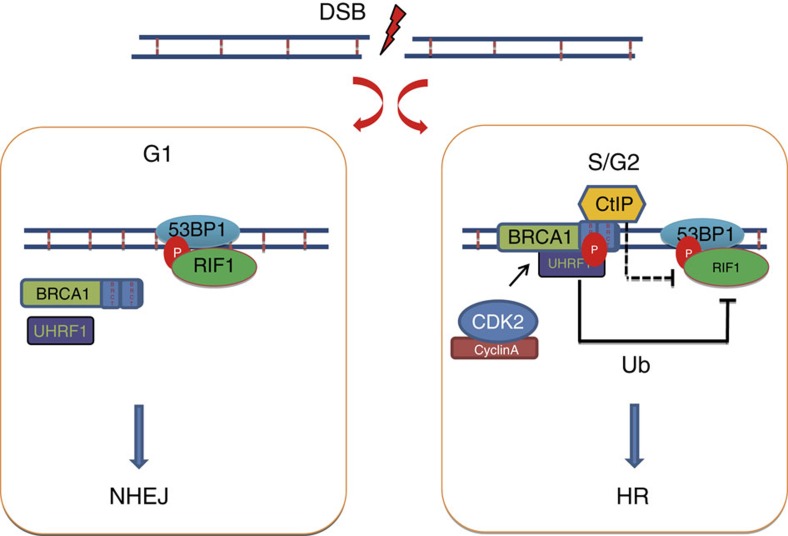
Model illustrating BRCA1-UHRF1 cascade regulating DNA repair choice.

Overall, our results suggest that UHRF1 is phosphorylated by CDK2/cyclin A at S674 in S phase, and is recruited to DSBs by BRCA1 through the recognizing of phosphorylated S674 by the BRCA1 BRCT domain. UHRF1 then mediates RIF1 ubiquitination, thereby inactivating RIF1 focus formation, facilitating the switch of DSB repair pathway choice from NHEJ to HR mediated by the BRCA1 pathway (Fig. 7).

## Discussion

Our data provided novel insights into the molecular basis by which BRCA1 antagonizes RIF1 in DNA repair in S phase. RIF1 acts as a key factor in the regulation of DSB repair pathway choice. RIF1 is recruited to DSB sites by interacting with ATM-phosphorylated 53BP1 (refs 49, 50, 51). In G1 phase, RIF1 blocks IR-induced BRCA1 foci and promotes NHEJ together with 53BP1. In S phase, RIF1 is removed from the DNA damage sites in a BRCA1-dependent manner, which facilitates HR. However, how BRCA1 antagonizes RIF1 function remained elusive. Given that RIF1 accumulation at DSBs requires the phosphorylation of 53BP1 by ATM^19^,^23^, it is conceivable that loss of 53BP1 phosphorylation in S phase might be one possible mechanism. However, 53BP1 phosphorylation remains comparable throughout S phase^52^, suggesting the removal of RIF1 from DSBs by BRCA1 relies on other mechanisms. CtIP has been implicated in the negative regulation of RIF1 foci in S/G2 phase^19^. However, another study shows that depletion of CtIP only partially affects RIF1 foci in G2 phase, but not in S phase^18^. In our study, we found the UHRF1 is sufficient to suppress RIF1 accumulation at DSBs in S phase and CtIP functions as a negative regulator of RIF1 only in G2 phase (Fig. 4e; Supplementary Fig. 3c,d). In G2 phase, both UHRF1 and CtIP are required for the functional suppression of RIF1 (Fig. 4e; Supplementary Fig. 3c,d). How UHRF1 cooperates with CtIP to regulate RIF1 function in G2 phase remains to be determined.

UHRF1 is highly correlated with cancer progression and metastasis^28^,^29^,^53^,^54^,^55^. However, the exact mechanism underlying the UHRF1-dependent tumorigenesis remains elusive. One possible explanation is based on the important role of UHRF1 in epigenetic regulation. UHRF1 was shown to recruit DNMT1 to replication forks to methylate the newly synthesized DNA and maintain the global genome methylation level^24^,^25^,^48^. Dysregulation of UHRF1 leads to the silencing of various tumour suppressor genes, which in turn trigger tumorigenesis^55^,^56^,^57^,^58^. Knockout UHRF1 in cancer cells induce complete cell cycle arrest^34^. Interestingly, the hypomorphic mutants (UHRF1^−/Neo^) with majority of UHRF1 depleted show normal cell proliferation^34^. UHRF1 is also important for development and cell proliferation of normal cells. UHRF1 knockout mice are embryonic lethal^33^. UHRF1 mutant zebrafish have global DNA hypomethylation, defective cell proliferation and embryonic defects^28^,^53^. Interestingly, UHRF1 overexpression in zebrafish also results in destablelized and mislocalization of DNMT1, DNA hypomethylation and cellular senescence^28^. Therefore, UHRF1 expression needs to be tightly regulated.

UHRF1 was also shown to be important for proper DDR^33^,^34^,^35^,^40^, although the molecular mechanism of its DDR function remained unclear. It is noteworthy that in a study using zebrafish as model, S661 (S661 of UHRF1 isoform 1 corresponds to S674 of isoform 2 in current study) of UHRF1 was also shown to be phosphorylated by CDK2/cyclin A, which regulate its cytoplasm/nuclear translocation^38^. We found that S674 in mammals does not affect UHRF1 nuclear localization. Although the site is conserved, the functional divergence might result from the interspecific differences.

Here we uncover the function of UHRF1 in DNA repair pathway choice is independent of its role in epigenetic regulation. Our identification of UHRF1 as a downstream effector of BRCA1 in regulating RIF1 provides a novel mechanism for UHRF1 function in the DDR. Intriguingly, many factors involved in the DDR are considered tumour suppressors, while UHRF1 is overexpressed in many cancers and considered oncogenic. It is possible that its role in epigenetic regulation is predominant in contributing to tumorigenesis when UHRF1 is overexpressed. It is also possible that overexpression could promote unscheduled DNA resection and promote genomic instability that leads to cancer formation. For instance, Rad51 overexpression promotes genomic instability and increased Rad51 expression has been observed in cancer^59^. BRCA1, when overexpressed, also increases DNA damage and genomic instability^60^. How UHRF1 function in the DDR contributes to tumorigenesis remains to be determined.

UHRF1 is composed of multiple functional domains including the UBL, TUDOR, PHD, SRA and RING domains. TUDOR, PHD and SRA domains have been reported to play important roles in epigenetic regulation^27^,^48^,^61^,^62^,^63^,^64^. The RING domain of UHRF1 is relatively less studied. A previous study suggests that the RING domain of UHRF1 is involved in DNMT1 degradation^65^,^66^. More recently, the RING domain has been shown to ubiquitinate histone H3K23 to facilitate global DNA methylation^46^. We found that UHRF1 mediates K63-linked polyubiquitination of RIF1, which promotes RIF1 dissociation from DSBs. The result that the UHRF1-S674A mutant does not regulate DNA methylation, gene expression, histone H3 monoubiquitination and only affect RIF1 ubiquitination indicates the regulation of RIF1 by UHRF1 is independent of UHRF1’s role in DNA replication and epigenetic regulation. Collectively, the results presented here have established a molecular mechanism connecting the UHRF1 E3 ubiquitin ligase activity and its function in maintaining genome stability, which is a new function for UHRF1 that is separate from its role in heterochromatin formation and epigenetics.

Many previous studies focused on UHRF1 isoform 1, although isoform 2 has also been studied^27^,^31^,^39^. Compared with UHRF1 isoform 1, UHRF1 isoform 2 only differs in the N terminus where it contains additional 13 amino acids. In our study, both of the two UHRF1 shRNAs target the sequences located within the conserved region. When we knocked down UHRF1 by shRNAs, both isoform 1 and isoform 2 levels should be decreased. When we reconstituted isoform 2 to UHRF1-depleted cells, UHRF1 isoform 2 could rescue previous reported isoform 1 function (such as DNA methylation, histone ubiquitination and transcription). Because the critical sites and domains required for UHRF1’s function in DNA repair exist in both isoforms, we believe that the findings of isoform 2 in our study can extend to isoform 1.

Elucidating the mechanisms for DSB repair pathway choice has important implications in understanding the pathogenesis of human diseases and cancer therapy. The identification of a BRCA1–UHRF1–RIF1 pathway highlights an intricate mechanism that regulates DSB repair pathway choice.

## Methods

### Cell lines

All cell lines used in this study were purchased from ATCC in 2014. The identities of all cell lines were confirmed by the medical genome facility at Mayo Clinic Center using short tandem repeat profiling upon receipt.

### Antibodies and constructs

The following antibodies were used in this study: UHRF1 (#612264, dilution: 1:1,000, BD Science) for western and IP and (H-8, dilution: 1:1000, Santa Cruz) for IF. BRCA1 (D-9, dilution: 1:100, Santa Cruz) for IF and (C-20, 1:200, Santa Cruz) for western blot and IP. RIF1 (A300-569A, dilution: 1:1,000, Bethyl Laborataries), HA (H9658, dilution: 1:1,000, Sigma), FLAG (F3165, dilution: 1:1,000, sigma), ubiquitin (SC-8017, dilution: 1:500, Santa Cruz), γ-H2AX (05-636, dilution: 1:500, Millipore), RPA (ab2175, dilution: 1:200, Abcam), RAD51(GTX70230, dilution: 1:200, GeneTex), 53BP1(NB100-304, dilution: 1:1,000, Novus), Phospho-(Ser) CDKs substrate antibody (9477s, dilution: 1:500, CST), cyclin A antibody (SC751, dilution: 1:200, Santa Cruz). CtIP (61141, dilution: 1:500, Active Motif). UHRF1, BRCA1 and RIF1 cDNAs were subcloned into the Flag-tagged vector (pIRES2-EGFP) HA-tagged vector (pCDNA3.1-HA or pCMV-HA). All mutants were generated by site-directed mutagenesis and confirmed by sequencing.

### RNAi target sequences

For siRNA transfection, cells were transfected twice at 24 h intervals with the indicated siRNA using Oligofectamine (Invitrogen) following the manufacturer’s instructions. The sequences of siRNAs against human BRCA1 (Thermo Fisher) were CAGCUACCCUUCCAUC-AUA and CUAGAAAUCUGUUGCUAUG. For lentiviral infection, shRNA lentiviral particles were packaged and transduced into the indicated cells according to the manufacturer’s guidelines (Sigma). The sequences of UHRF1 shRNAs were: #21 GCCTTTGATTCGTTCCTTCTT and #18 GCAAGAAGAAGGCGAAGATAA. The sequence of RIF1 shRNA was TCTTATGAGACGTATAGTATT. The sequence of 53BP1 shRNA was: AGAACGAGGAGACGGTAATAG. The sequence of BRCA1 shRNA was: TATAAGACCTCTGGCATGAAT. The sequence of DNMT1 shRNA was: TTGAATCTCTTGCACGAATTT. The sequence of UBC13 shRNA was: GCCTTGTTAAGTGCTCCCAAT. All the shRNAs were obtained from Sigma.

### Inducible system for UHRF1 knockdown

The UHRF1 shRNA oligonucleotides were annealed and ligated into *Age*I/EcoRI-linearized Tet-on-pLKO.1 lentiviral vector (Addgene). For the induced UHRF1 knockdown in synchronized cells, HeLa cells stably expressing Tet-on-shRNA targeting UHRF1 were treated with doxycycline (1 μg ml^−1^) for 18 h before the second round of release of the double thymidine block to ensure the downregulation of UHRF1 levels in released cells^67^.

### Immunoprecipitation and GST-pull-down assay

Cells were lysed with NETN buffer (20 mM Tris-HCl (pH 8.0), 100 mM NaCl, 1 mM EDTA and 0.5% NP-40) containing protease inhibitors on ice for 30 min. Following sonication, cell lysates were clarified by centrifugation and incubated with protein G or protein A agarose beads coupled with antibody against the indicated proteins for 8 h at 4 °C. Beads were then washed with NETN buffer three times and analysed by western blot. For tagged protein IP, cell lysates were incubated with Anti-Flag M2 Affinity beads (Sigma) for 3 h at 4 °C, EZview Red anti-HA affinity beads (Sigma). Precipitates were then washed and immunoblotted with the indicated antibodies. For the BRCA1 GST-pull-down assay, GST-BRCA1 fragments fusion proteins were expressed in *Escherichia coli*. Purified fusion proteins were immobilized on glutathione Sepharose 4B beads and incubated with cell lysates at 4 °C. The samples were separated by SDS–polyacrylamide gel electrophoresis (PAGE) and analysed by western blot. The uncropped versions of western blots are shown in Supplementary Fig. 7.

### Far-western ligand blots

Far-western ligand blot was performed in TBST buffer supplemented with 5% skim milk powder. Following *in vitro* kinase assay, the His-UHRF1 (WT or S674A) were subjected to SDS–PAGE and transferred to membranes. After the denaturation/renaturation procedure using guanidine hydrochloride, the blot was incubated overnight at 4 °C with 2 μg ml^−1^ of GST-BRCT (WT or S1655A respectively). Bound protein was detected with purified antibody against GST.

### DNA repair assay

Integrated DNA repair reporter systems were used to determine the HR and NHEJ efficiency^41^. Briefly, HEK293 cells integrated with HR or NHEJ reporters were infected with the indicated viruses. Forty-eight hours after infection, 4-Hydroxytamoxifen (4OHT) was added at 3 mM for 24 h. Three days after 4OHT was added, the percentage of GFP-positive cells was analysed by FACS^41^. HR efficiency is presented as the percentage of control cells. Repair frequencies are the mean of at least three independent experiments and error bars represent the s.d. from the mean value. Statistical analysis was performed by the Student’s *t-*test for two groups and by ANOVA for multiple groups. *P*<0.05 was considered significant.

### Immunofluorescence staining

Cells cultured on coverslips were treated with IR followed by recovery for the indicated times. After washing with PBS, cells were fixed in 3% paraformaldehyde for 15 min and permeabilized in 0.5% triton X-100 solution for 5 min at room temperature. Cells were blocked with 5% goat serum and incubated with primary antibody for 60 min. Subsequently, samples were washed and incubated with secondary antibody for 60 min. DAPI staining was performed to visualize nuclear DNA. The coverslips were mounted onto glass slides with anti-fade solution and visualized using a Nikon ECLIPSE E800 fluorescence microscope.

### *In vivo* ubiquitination assay

Transfected HEK 293T cells were irradiated (10 Gy). After 1 h, cell lysates were prepared with 120 ml of 62.5 mM Tris-HCl (pH 6.8), 2% SDS, 10% glycerol, 20 mM NEM and 1 mM iodoacetamide, boiled for 15 min, diluted 10 times with NETN buffer containing protease inhibitors, 20 mM NEM, and 1 mM iodoacetamide, and clarified by centrifuge (16,000*g*, 10 min, 4 °C). The lysates were immunoprecipitated with the indicated antibody at 4 °C with agitation. The precipitates were eluted in SDS sample buffer and analysed by western blot with the indicated antibodies.

### *In vitro* ubiquitination assay

The Flag-RIF1 or 9KR mutant was stably expressed in HEK 293T cells and purified by immunoprecipitation with anti-Flag M2 beads (Sigma). The proteins were then eluted with 3 × FLAG Peptide (Sigma). The recombinant His-UHRF1 or H754A mutant was expressed in *E*. *coli* and purified with a His-tag purification column (Novagen). *In vitro* ubiquitination assays were performed with 300 ng of ubiquitin-activating enzyme (UBE1) (Boston Biochem), 200 ng of purified UBC13/MMS2 (Boston Biochem), 2 μg of Myc-ubiquitin (Boston Biochem), 3 μg His-UHRF1 or H754A mutant in 40 μl of reaction buffer (50 mM Tris (pH 7.5), 2.5 mM MgCl_2_, 2 mM ATP, and 2 mM DTT). The reactions were carried out at 37 °C for 45 min, and stopped by boiling in SDS sample buffer.

### Laser microirradiation

A customized laser microirradiation system consisting of an inverted microscope (Nikon), a laser ablation unit (Photonic Instruments) and microscope automation and imaging software (Metamorph, Molecular Devices) were used^68^,^69^. Briefly, a 337 nm nitrogen laser (with 1–20 Hz repetition rate, 2–6 ns pulse duration and 120 mJ per pulse energy) transmits radiation through an optical fibre and a dye cell containing a solution that produces a 551-nm dye laser. The laser microbeam is then focused by a 633 (numerical aperture (NA) 1.4) oil immersion microscope objective. The total laser energy delivered to each focused spot was set by an attenuator plate (50% transmission) and the number of pulses. Cells were cultured on 35 mm glass-bottomed dishes before laser irradiation. Following laser irradiation, cells were fixed with 4% paraformaldehye for 10 min at room temperature. Immunofluorescence staining was performed and cells were then imaged using the Nikon microscope and the MetaMorph software.

### *In vitro* kinase assays

Active CDK2/cyclin A (14–448) was purchased from Millipore. Kinase assays were performed in the presence of (γ-32P) ATP by using an *in vitro* kinase buffer system from Millipore. Briefly, recombinant CDK and cyclin complexes were incubated with various substrates at 30 °C for 40 min. The reaction samples were subjected SDS–PAGE and autoradiography.

### Cell cycle synchronization

Cell cycle synchronization was performed with minor modifications. Briefly, cells were treated with 2 mM thymidine for 17 h and released in fresh medium for another 9 h. Following the second block by thymidine (2 mM, 18 h), cells were collected as G1-phase samples (unreleased) and S-phase samples (2.5 or 5 h after release into fresh medium without thymidine) respectively followed by irradiation and analysed accordingly.

### Tandem affinity purificationchromatin associated extraction

Cells were lysed with NETN buffer (100 nM NaCl) on ice for 20 min. After centrifugation, supernatant (soluble fraction) were discarded and the pellet was resolved in NETN buffer with sonication (chromatin fraction). Afterwards, the chromatin fraction was incubated with streptavidin sepharose beads for 4 h at 4 °C. The bead-bound proteins were washed three times with NETN buffer and eluted twice with 2 mg ml^−1^ biotin (Sigma-Aldrich) for 1 h at 4 °C. The eluates were combined and then incubated with S-protein agarose (Novagen) for 4 h at 4 °C. The S-protein agarose beads were washed three times with NETN buffer. The proteins bound to S-protein agarose beads were separated by SDS–PAGE and visualized by Coomassie Blue staining. HEK 293T cells expressing SFB-BRCA1 were used for BRCA1 purification. For RIF1 purification, endogenous RIF1 were purified in HCT116 cells with tandem affinity purification.

### Mass spectrometry

After staining proteins in SDS–PAGE gels with Coomassie blue, gel lanes were sliced into different bands and in-gel digested overnight at 37 °C with trypsin. After digestion, peptides were extracted twice in 200 μl of acetonitrile with re-suspension in 20 μl of 2% formic acid prior to second extraction, dried in a Savant SpeedVac, and dissolved in a 5% methanol/0.1% formic acid solution. Tryptic peptides were separated on a C18 column, and were analysed by LTQ-Orbitrap Velos (Thermo). Proteins were identified by using the National Center for Biotechnology Information search engine against the human or mouse RefSeq protein databases.

### BIAcore peptide binding assay

Surface plasmon resonance measurements were performed on BIAcore TP200 (BIAcore). Biotinylated peptides (non-phosphorylated S674 peptide: PSRAGSPRRTSKKTKVK-Biotin, phosphorylated pS674 peptide: PSRAG(pS)PRRTSKKTKVK-Biotin) were passed over the surface of the streptavidin-coated chip(GE 29104992) to an equivalent around 250 resonance units (RU). Proteins were dialyzed in buffer containing 150 mM NaCl, 0.005% (w/v) Polysorbate 20 and 10 mM HEPES, pH 7.4 and 1 mM PMSF before BIAcore analysis. Analytes were diluted with the same buffer to achieve concentrations from 39 nM to 2.5 μM. Analytes (90–150 μl) were injected at a speed of 30 μl min^−1^. To regenerate the surface after each sample, 10 μl of 0.005% SDS in 50 mM Tris-HCl (pH 7.5), 0.15 M NaCl, and 1 mM PMSF was injected twice at a rate of 10 μl min^−1^.

### Microarray analysis

Transcriptome profiles were measured using Affymetrix HG-U133_Plus_2 arrays. Pre-processing and normalization was done using the Partek microarray data analysis software (http://www.partek.com/). Partek pre-processes raw intensity files from microarray experiment using RMA’s background subtraction and uses quantile normalization as the normalization technique. Principle Component Analysis and unsupervised hierarchical clustering were used to perform quality checks on the samples. The samples from different groups were compared using 2-way ANOVA (analysis of variance). Significantly changed genes obtained from ANOVA were prioritized by a combination of *P* value and fold change.

### Statistical analysis

The statistical data were from three biological triplicates. Statistical analysis was performed by the Student’s *t*-test for two groups and by ANOVA for multiple groups. *P*<0.05 was considered significant.

## Additional information

**Accession codes:** Microarray data have been deposited in Gene Expression Omnibus database under accession code GSE73751.

**How to cite this article:** Zhang, H. *et al.* A cell cycle-dependent BRCA1–UHRF1 cascade regulates DNA double-strand break repair pathway choice. *Nat. Commun.* 7:10201 doi: 10.1038/ncomms10201 (2016).

## Supplementary Material

Supplementary Information Supplementary Figures 1-7

Supplementary Data 1 Analysis summary of microarray data
