# Supplementary material for: A cell cycle-dependent BRCA1–UHRF1 cascade regulates DNA double-strand break repair pathway choice
Source: Nat Commun. 2016 Jan 5;7:10201. doi: 10.1038/ncomms10201 (PMC4728409; doi:10.1038/ncomms10201)
Supplement: Supplementary Information — Supplementary Figures 1-7 [file ncomms10201-s1.pdf]

Supplementary Figure 1

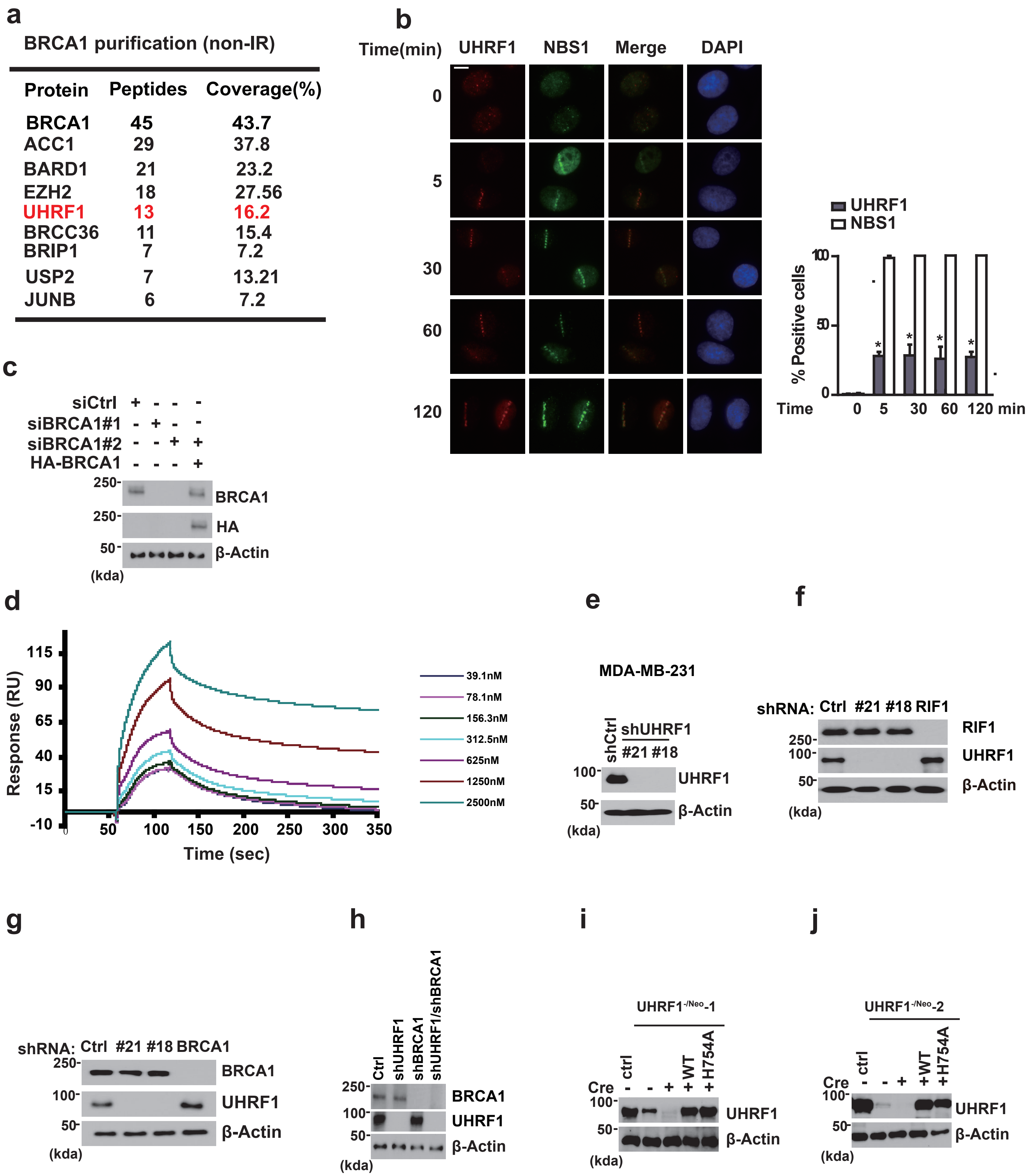

Supplementary Figure 1

UHRF1 interacts with BRCA1 and is recruited to DNA damage sites by BRCA1 in S phase.

(**a**) Tandem affinity purification was performed using 293T cells stably expressing SBP-tagged BRCA1 before DNA damage. Selective hits from mass spectrometry analysis were shown in the table. This is related to Fig. 1a, which shows DNA damage treated samples**.**

(**b**) Kinetics analysis of UHRF1 recruitment to DNA damage sites. U2OS cells were subjected to laser micro-irradiation to generate DSBs in a line pattern. Cells were then fixed and immunostained with the NBS1 and UHRF1 antibodies at the indicated time points. For each condition, 200 cells were counted. Error bars represent the mean ± SD of 3 biological triplicats. UHRF1 recruitment positive cell percentage compared with control group: * $P < 0.05$. Scale bar, 10um.

(**c**) SPR of immobilized pS674 peptide exposed to increasing concentrations of purified GST-BRCA1-BRCT domain.

(**d**) Immunoblot with the indicated antibodies for samples in Fig. 3a-b.

(**e-g**) Immunoblot with the indicated antibodies for samples in Fig. 3c-d

(**h-i**) Immunoblot with the indicated antibodies for samples in Fig. 3e

Supplementary Figure 2

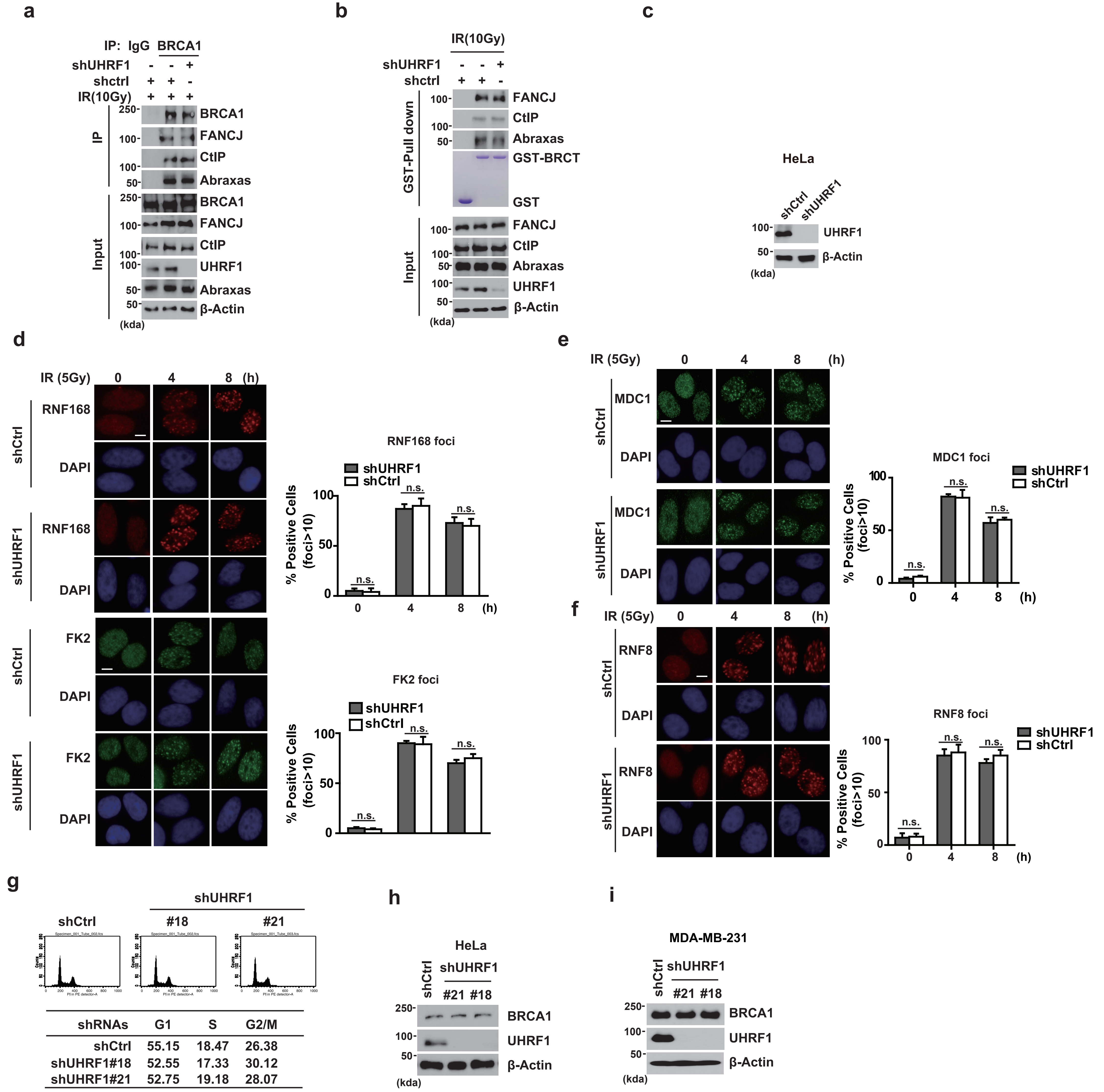

shRNAs	G1	S	G2/M
shCtrl	55.15	18.47	26.38
shUHRF1#18	52.55	17.33	30.12
shUHRF1#21	52.75	19.18	28.07

Supplementary Figure 2

UHRF1 is involved in DNA repair through its E3 ligase activity.

(**a**) co-immunoprecipitation (Co-IP) between BRCA1 and indicated proteins in HeLa cells was performed following IR (10Gy).

(**b**) GST pull-down assay of indicated proteins using wild-type BRCA1 BRCT domain following IR (10Gy)

(**c**) Immunoblot with the indicated antibodies for samples in Fig. 3f and supplementary Fig. 2d-f.

(**d-f**) HeLa cells stably expressing UHRF1 shRNA were irradiated and fixed at indicated time points. Focus formation for the RNF168, FK2 (d), MDC1 (**e**) or RNF8 (**f**) were examined as indicated. Quantification for positive cells (foci>10/cell) was shown on the right panel. For each sample, randomly selected 600 cells were counted. Data presented as mean ± SD of 3 biological triplicates. Positive cell percentage compared with control group. n.s: no siginficant difference. Scale bar, 10um.

(**g**) Cell cycle profile analysis for UHRF1 knockdown. Immunoblot with the indicated antibodies for samples in Fig. 3f.

(**h**) Immunoblot with the indicated antibodies for HeLa cells cell lysates.

(**i**) Immunoblot with the indicated antibodies for MDA-MB-231 cells cell lysates.

Supplementary Figure 3

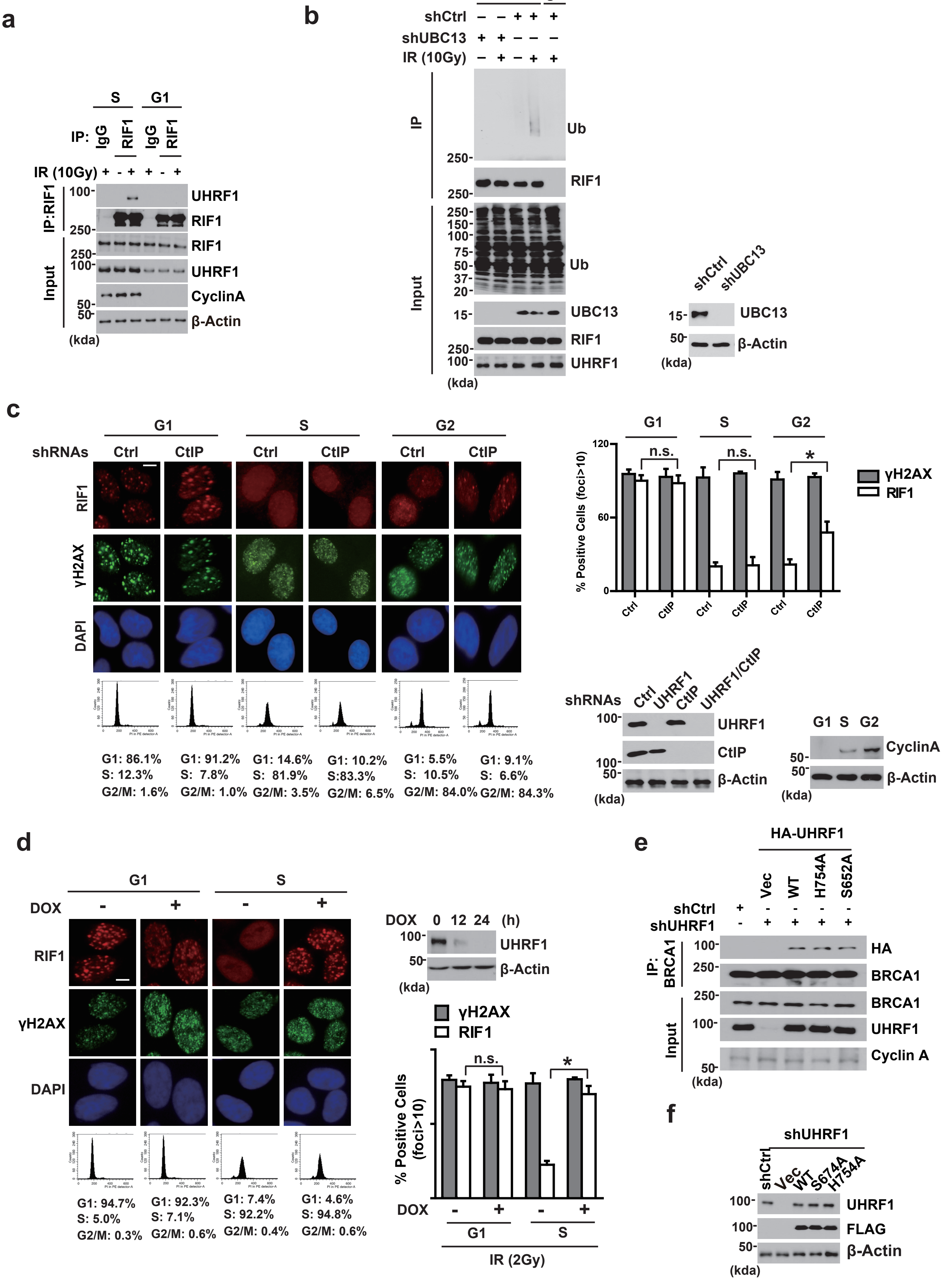

Supplementary Figure 3

UHRF1 ubiquitinates RIF1.

(**a**) The interaction between UHRF1 and RIF1 is induced by IR in S phase. HeLa cells were synchronized in S phase or G1 phase with or without IR treatment, and the UHRF1-BRCA1 interaction was examined by co-immunoprecipitation (Co-IP) and western blot.

(**b**) UBC13 is important for UHRF1-dependent Lys63-linked poly-ubiquitination of RIF1 *in vivo*. RIF1 ubiquitination was examined in HeLa cells expressing indicated shRNAs.

(**c**) CtIP promotes removal of RIF1 from DSBs in G2 phase but not S phase. HeLa cells stably expressing the indicated shRNAs were synchronized and irradiated (2 Gy). RIF1 IRIF was examined. Upper left: Representative micrographs. Scale bar, 10um. Upper right: Quantitation of the positive cells (foci>10/cell) as indicated. For each condition, randomly selected 600 cells were counted. Data presented as mean ± SD of 3 biological triplicates. RIF1 foci Positive cell percentage compared with control group. * $P<0.05$. n.s: no siginficant difference. Lower left: cell cycle profiles of synchronized samples. Lower right: immunoblot of lysates with indicated antibodies.

(**d**) RIF1 focus formation was examined in cells depleted of UHRF1 using Doxycycline (Dox)-induction in S phase (See Methods). Upper left: Representative micrographs. Scale bar, 10um. Upper right: immunoblot of lysates with the indicated antibodies. Lower left: cell cycle profiles of synchronized samples. Lower right: quantitation of the positive cells (foci>10/cell) as indicated. For each sample, randomly selected 600 cells were counted. Data presented as mean ± SD of 3 biological triplicates. RIF1 foci Positive cell percentage compared with control group. * $P<0.05$. n.s: no siginficant difference.

(**e**) UHRF1 H754A mutant does not affect the UHRF1-BRCA1 interaction. HEK293T cells stably expressing UHRF1 shRNA were reconstituted with indicated constructs. The UHRF1-BRCA1 interaction was examined by co-immunoprecipitation (Co-IP) and western blot.

(**f**) Immunoblot with the indicated antibodies for the HeLa cell lysates in Fig. 5c

Supplementary Figure 4

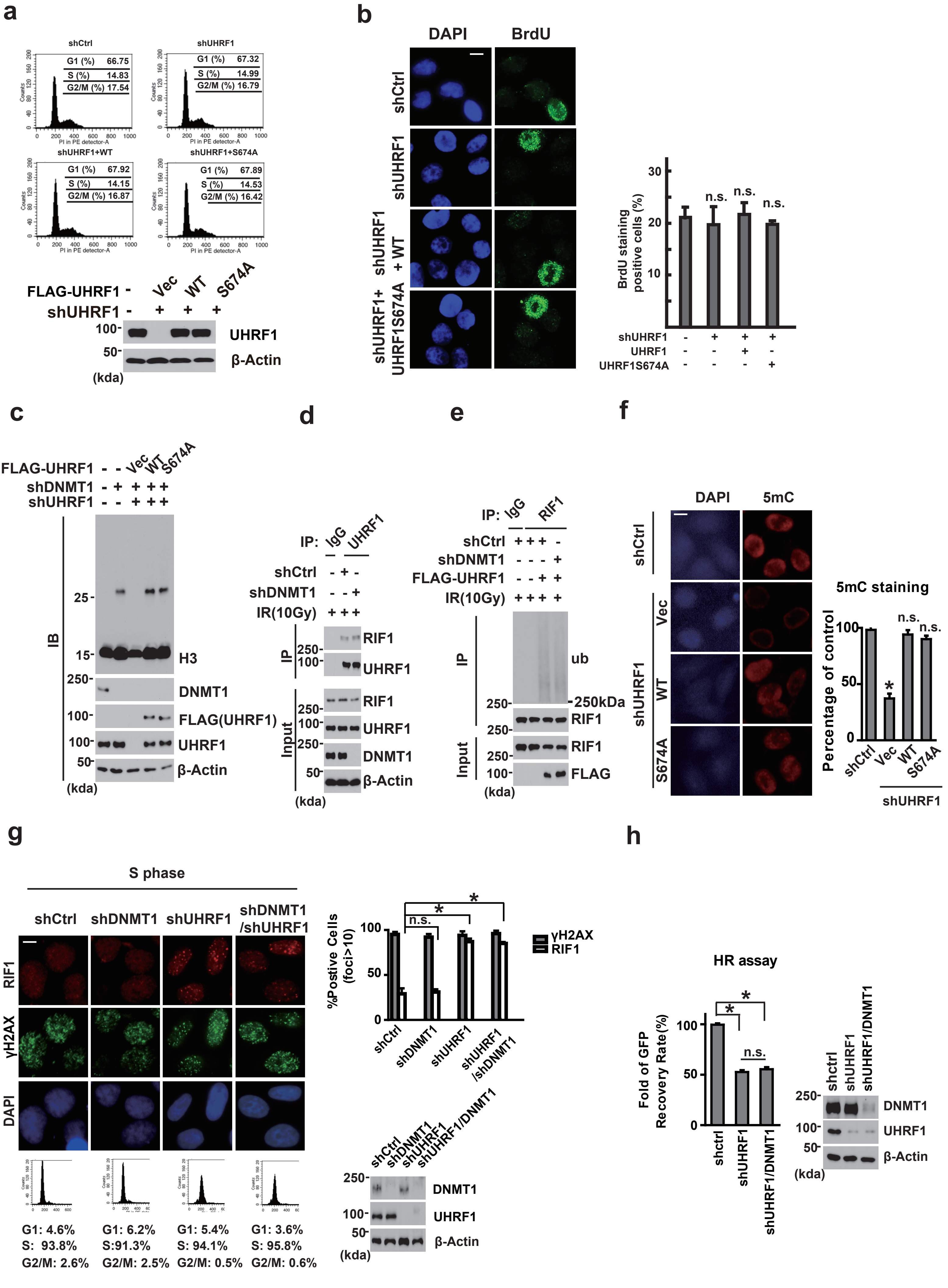

Supplementary Figure 4

UHRF1 Ser674 phosphorylation is important for RIF1 ubiquitination.

(**a**) Cell-cycle profiles of HeLa cells with the indicated treatments were examined by FACS.lower panel: Immunoblot samples in (a) and (b) with indicated antibodies.

(**b**) BrdU incorporation was investigated in the indicated cells. Left: representative micrographs. Scale bar, 10um. Right: quantitation of the positive cells as indicated. For each sample, randomly selected 600 cells were counted. Data presented as mean ± SD of 3 biological triplicates. Positive cell percentage compared with control group. n.s: no siginficant difference.

(**c**). Ser 674 phosphorylation is not important for UHRF1 mediated H3 ubiquitination. Endogenous DNMT1 and/or UHRF1 were knocked down using shRNAs in HeLa cells expressing FLAG-tagged UHRF1 (WT or S674A mutant). Cells were collected 72 hrs following knockdown and the cell lysates were subject to immunoblotting with the indicated antibodies.

(**d**) UHRF1-RIF1 interaction is independent of DNMT1. HeLa cells stably expressing control or DNMT1 shRNA were irradiated (10Gy). The UHRF1-RIF1 interaction was then examined by Co-IP.

(**e**) UHRF1 was transfected into HeLa cells stably expressing control or DNMT1 shRNA. RIF1 ubiquitination was then examined following irradiation (10Gy).

(**f**) 5mC staining were performed as indicated treatments. Left: representative image. Scale bar, 10um; Right: quantitation of the positive cells as indicated. For each sample, randomly selected 200 cells were counted. Data presented as mean ± SD of 3 biological triplicates. Positive cell percentage compared with control group. *p<0.05. n.s: no siginficant difference.

(**g**) rH2AX and RIF1 foci formation were detected with indicated treatments. Left upper: representative images. Scale bar, 10um; Left lower: cell cycle profile; Right upper: quantification of positive cells as indicated. For each sample, randomly selected 200 cells were counted. Data presented as mean ± SD of 3 biological triplicates. RIF1 foci Positive cell percentage compared with control group. *p<0.05. n.s: no siginficant difference.

(**h**) HR assay were performed with the indicated treatments. Left: quantification as indicated; Right: immunoblot for the cell lysates. Data presented as mean ± SD of 3 biological triplicates. Positive cell percentage compared with control group. *p<0.05. n.s: no siginficant difference.

Supplementary Figure 5

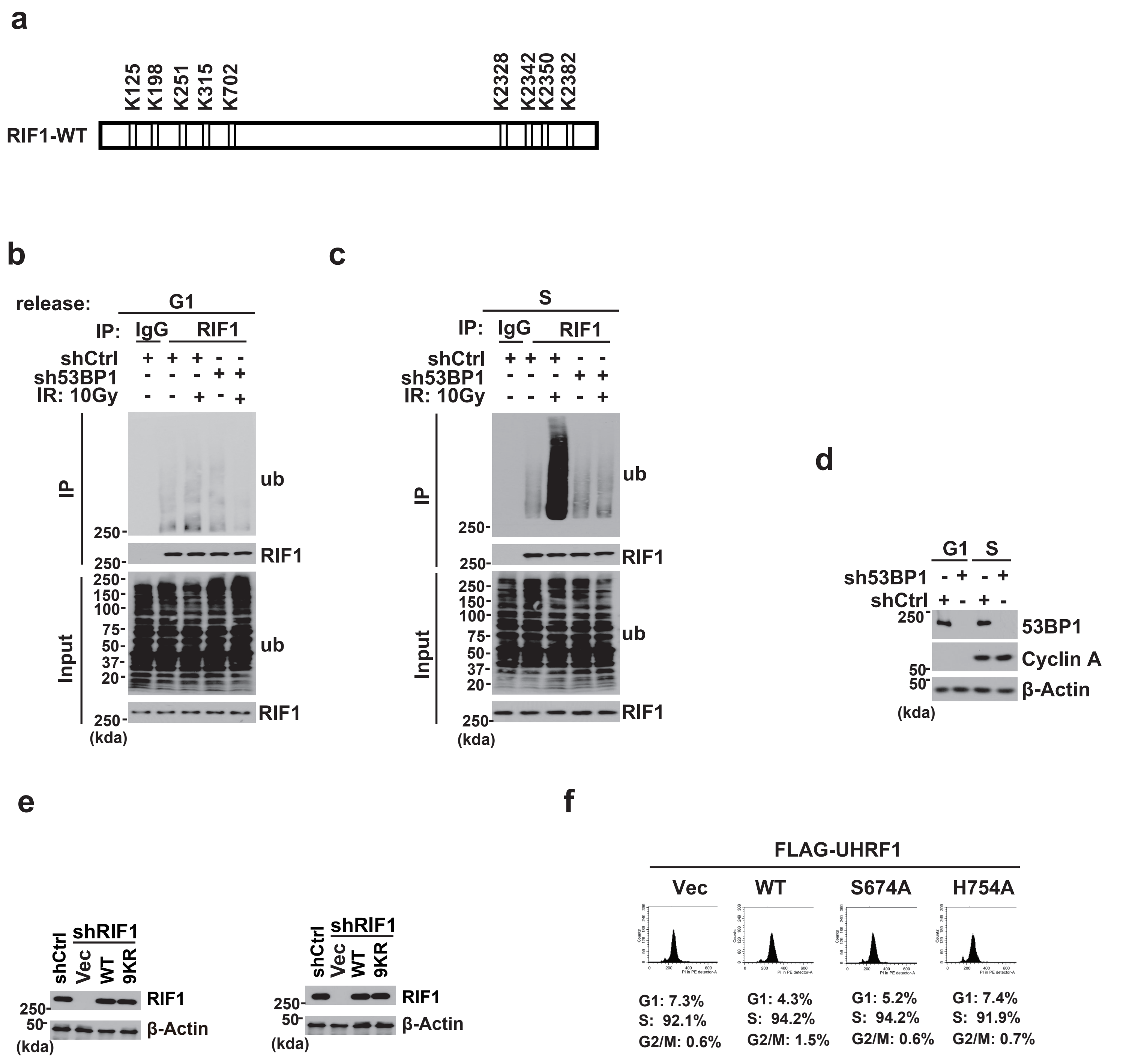

Supplementary Figure 5

RIF1 ubiquitination by UHRF1 is important for its accumulation at DSB sites.

(**a**) Schematic diagram of RIF1 ubiquitination sites.

(**b-c**) 53BP1 is important for RIF1 ubiquitination. HeLa cells stably expressing indicated shRNAs were synchronized at G1 or S phase. RIF1 ubiquitination was then examined following irradiation (10Gy).

(**d-e**) Western blot for samples in Fig. 6g-h.

(**f**) cell-cycle profile for the Fig. 6f.

Supplementary Figure 6

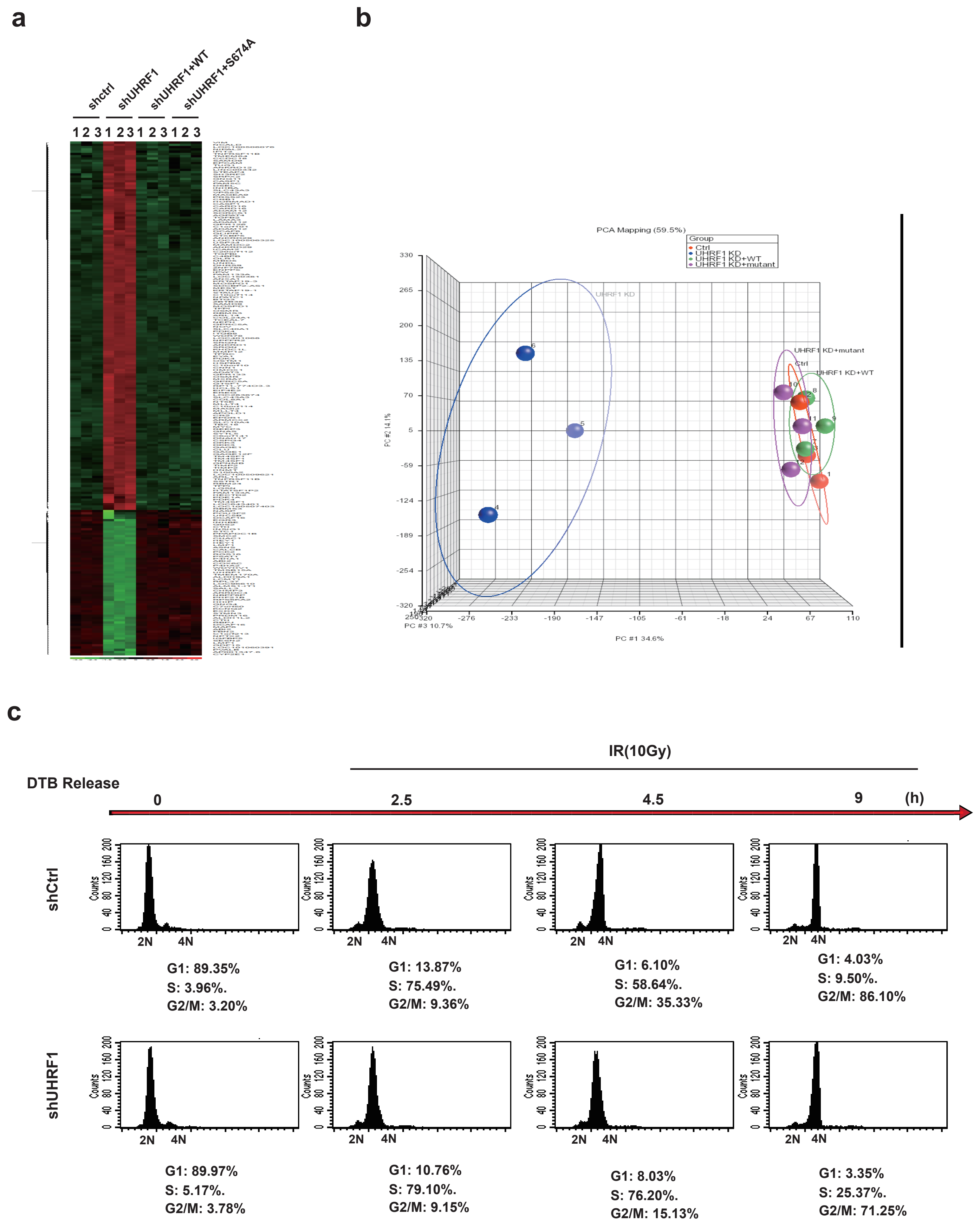

Supplementary Figure 6

(a-b) Microarray analysis were performed as describe in Methods. (**a**) Heatmap for the gene profiling analysis. (**b**) PCA analysis.

(c) HeLa cells stably expressing control or UHRF1 shRNA were synchronized with double thymidine block. FACS profile were examined as indicated.

Figure 1

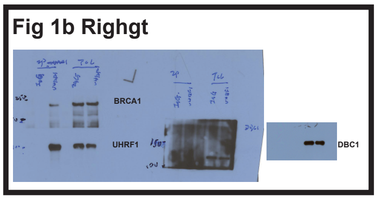

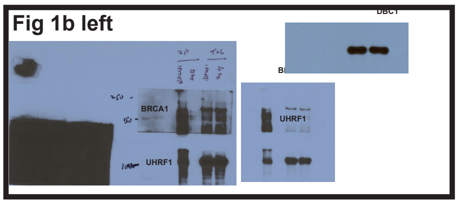

Figure 2

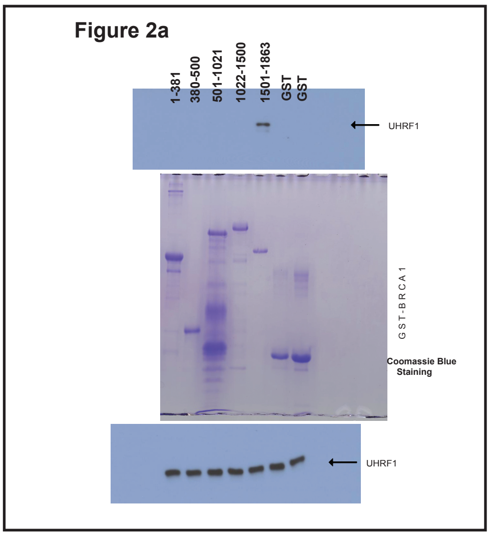

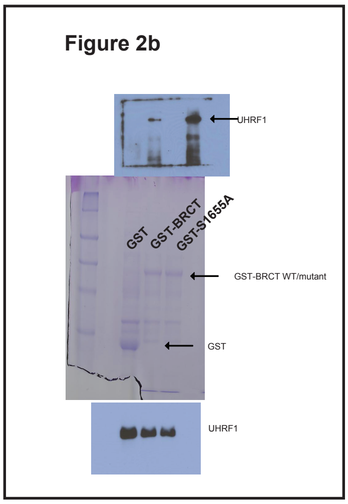

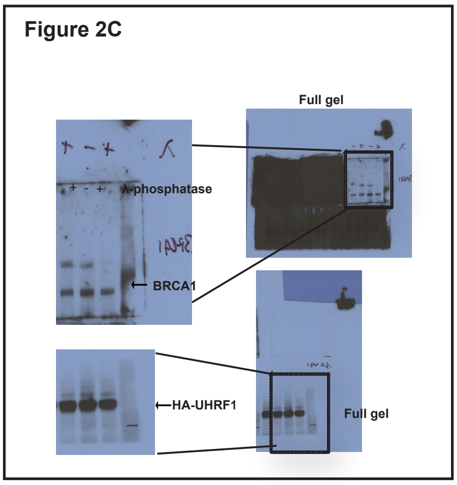

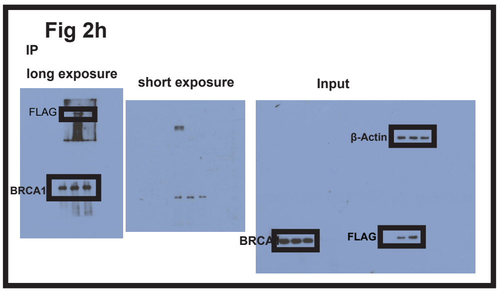

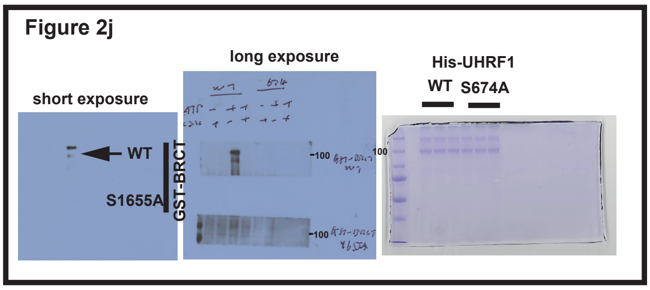

Figure 4

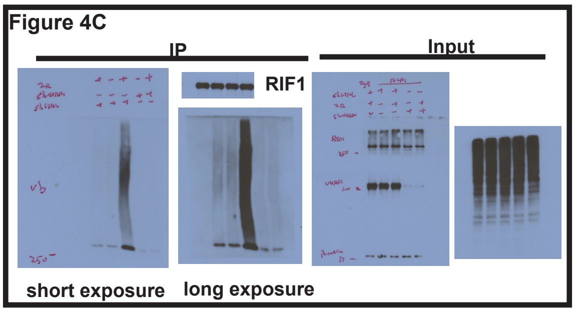

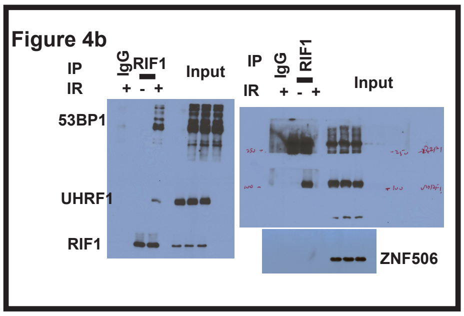

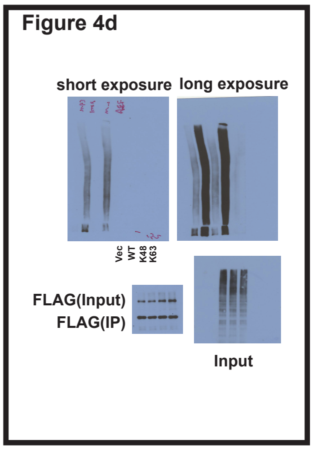

Figure 5

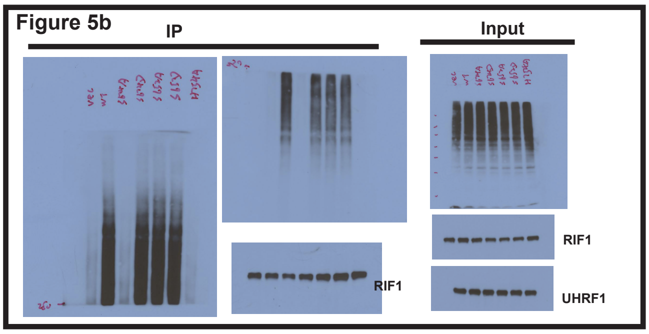

Figure 6

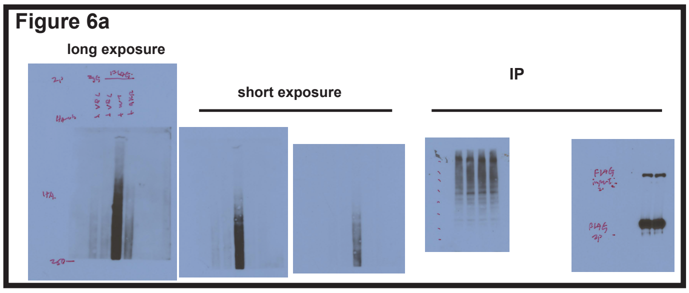

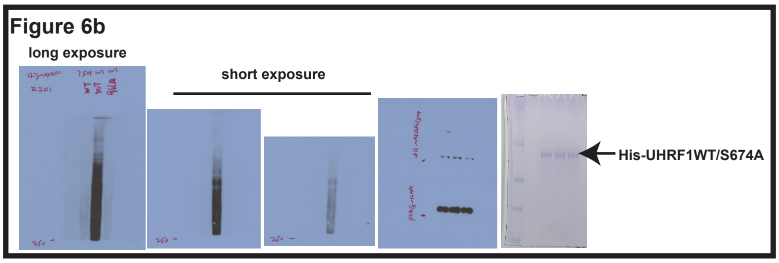

Supplementary Figure 7 uncroped main blots
